# Supplementary figures and images for: Phosphatidylcholine mediates the crosstalk between LET-607 and DAF-16 stress response pathways
Source: PLoS Genet. 2021 May 20;17(5):e1009573. doi: 10.1371/journal.pgen.1009573 (PMC8172019; doi:10.1371/journal.pgen.1009573)

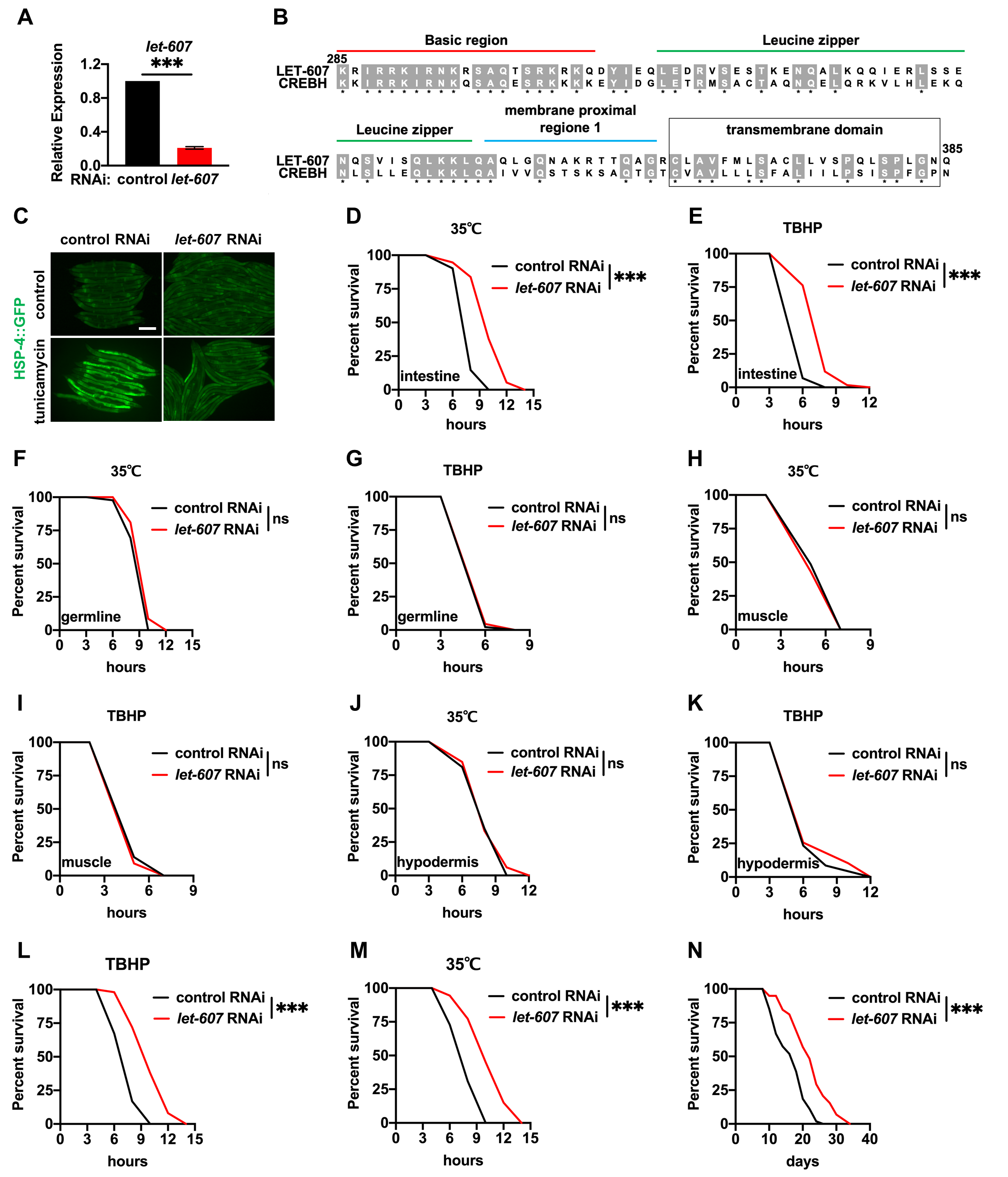

Supplement: S1 Fig — (A) mRNA levels of let-607 in let-607 RNAi-treated worms (1:5 diluted) as measured by qPCR. n = 3 per group. (B) Comparison of LET-607 and CREBH protein sequences. (C) Effects of let-607 RNAi on HSP-4::GFP expression induced by tunicamycin. Scale bar = 100 μm. (D-K) Effects of tissue-specific let-607 RNAi on TBHP and heat stress resistance. (L-M) Effects of post-developmental let-607 RNAi on TBHP resistance (L), heat resistance (M) and lifespan (N). Data were presented as mean ± SEM. *** p < 0.001. (TIF) [file pgen.1009573.s001.TIF]

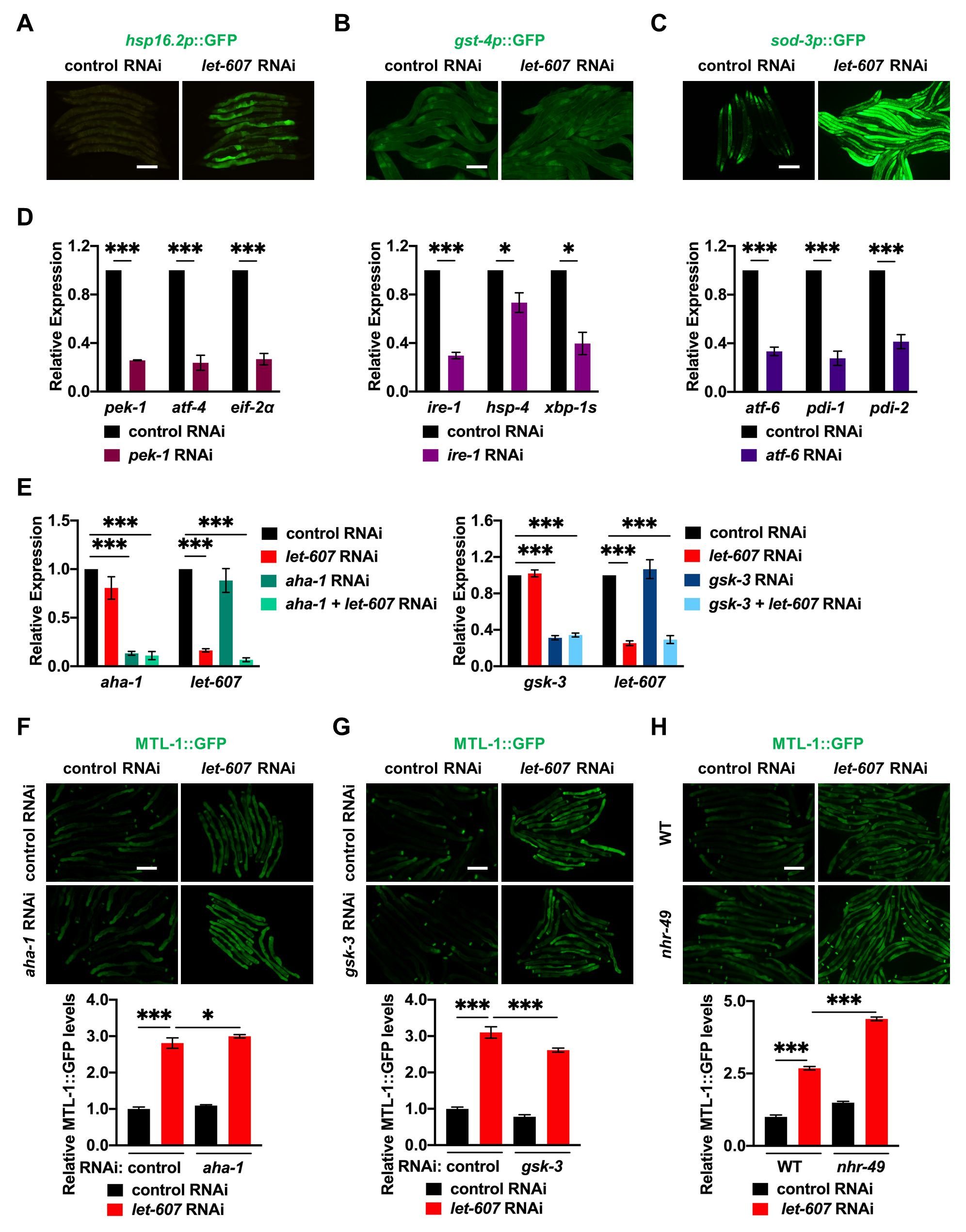

Supplement: S2 Fig — (A-C) Effects of let-607 RNAi on the expression of hsp-16.2p::GFP (A), gst-4p::GFP (B) and sod-3p::GFP (C). Scale bar = 100 μm. (D) Effects of ER UPR genes RNAi on their own expression and downstream targets. n = 3 per group. (E) Knockdown efficiencies of aha-1 and gsk-3 RNAi. n = 3 per group. (F-H) Effects of aha-1 RNAi (F), gsk-3 RNAi (G) and nhr-49 mutation (H) on MTL-1::GFP expression. Upper panel, representative images. Lower panel: quantification data. Scale bar = 100 μm. Data were presented as mean ± SEM. * p < 0.05, *** p < 0.001. (TIF) [file pgen.1009573.s002.TIF]

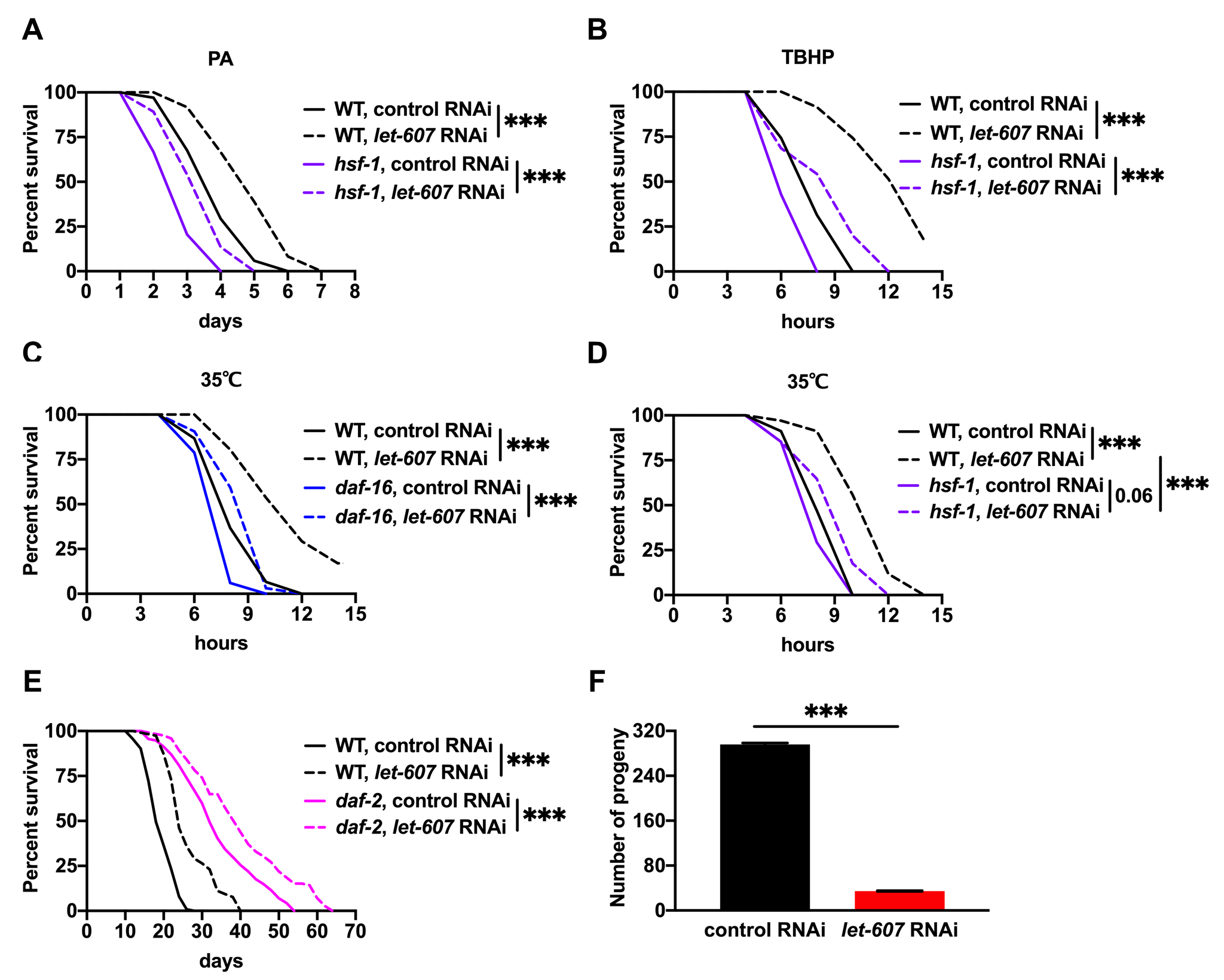

Supplement: S3 Fig — (A-B) Impact of let-607 RNAi on pathogen resistance (A) and TBHP resistance (B) in WT and hsf-1 mutant worms. (C-D) Effects of let-607 RNAi on heat stress resistance in daf-16 (C) and hsf-1 (D) mutants. (E) Impact of let-607 RNAi on the lifespan of daf-2 mutants. (F) Effects of let-607 RNAi on reproduction. Data were presented as mean ± SEM. *** p < 0.001. (TIF) [file pgen.1009573.s003.TIF]

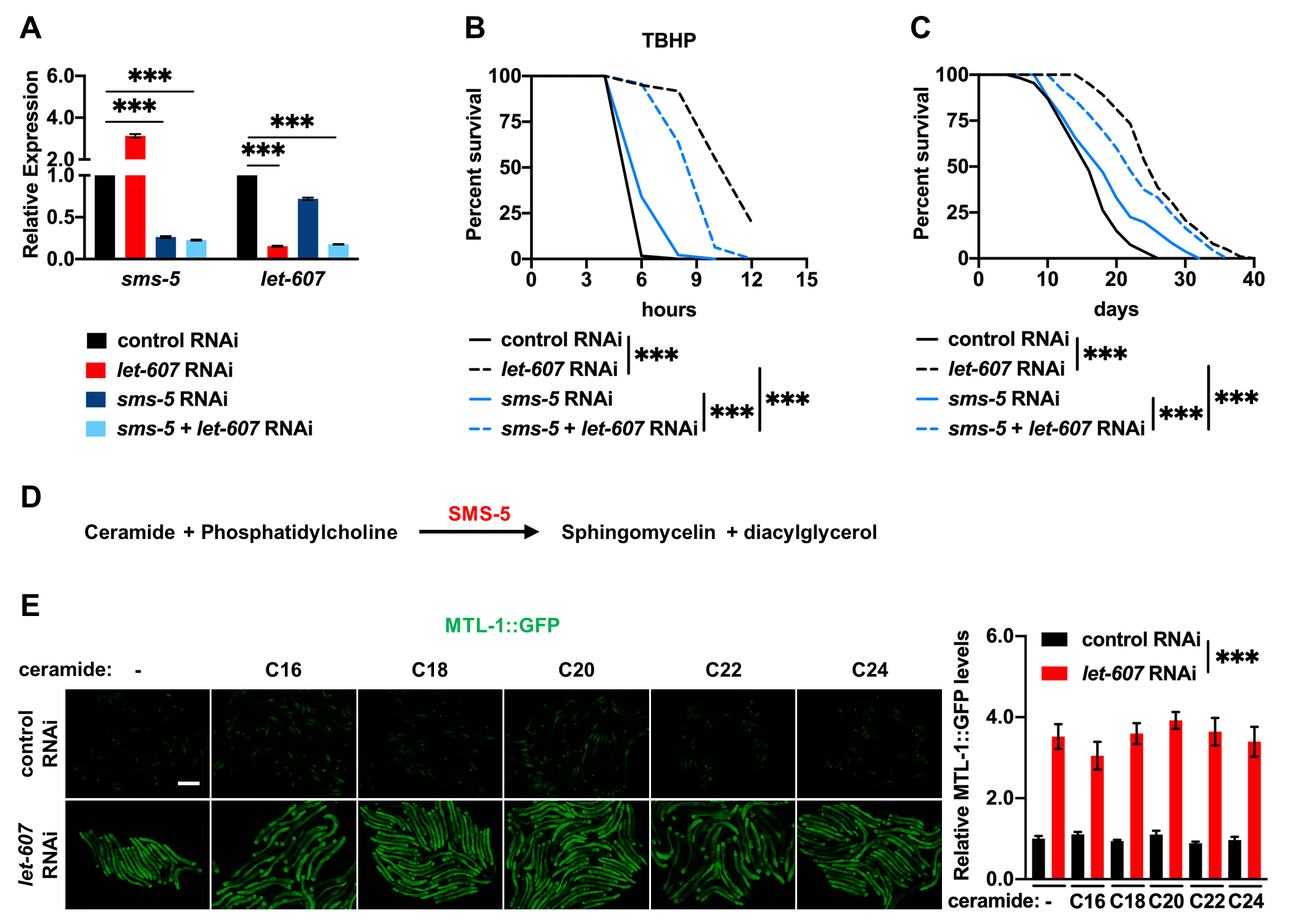

Supplement: S4 Fig — (A) Knockdown efficiency of sms-5 RNAi. n = 3 per group. (B-C) Effects of sms-5 RNAi on let-607 RNAi-induced TBHP resistance (B) and longevity (C). (D) The schematic of SMS-5-mediated enzymatic reaction. (E) Supplementation of ceramides did not influence MTL-1::GFP expression with or without let-607 RNAi. Left panel, representative images. Right panel: quantification data. Scale bar = 100 μm. Data were presented as mean ± SEM. *** p < 0.001. (TIF) [file pgen.1009573.s004.TIF]

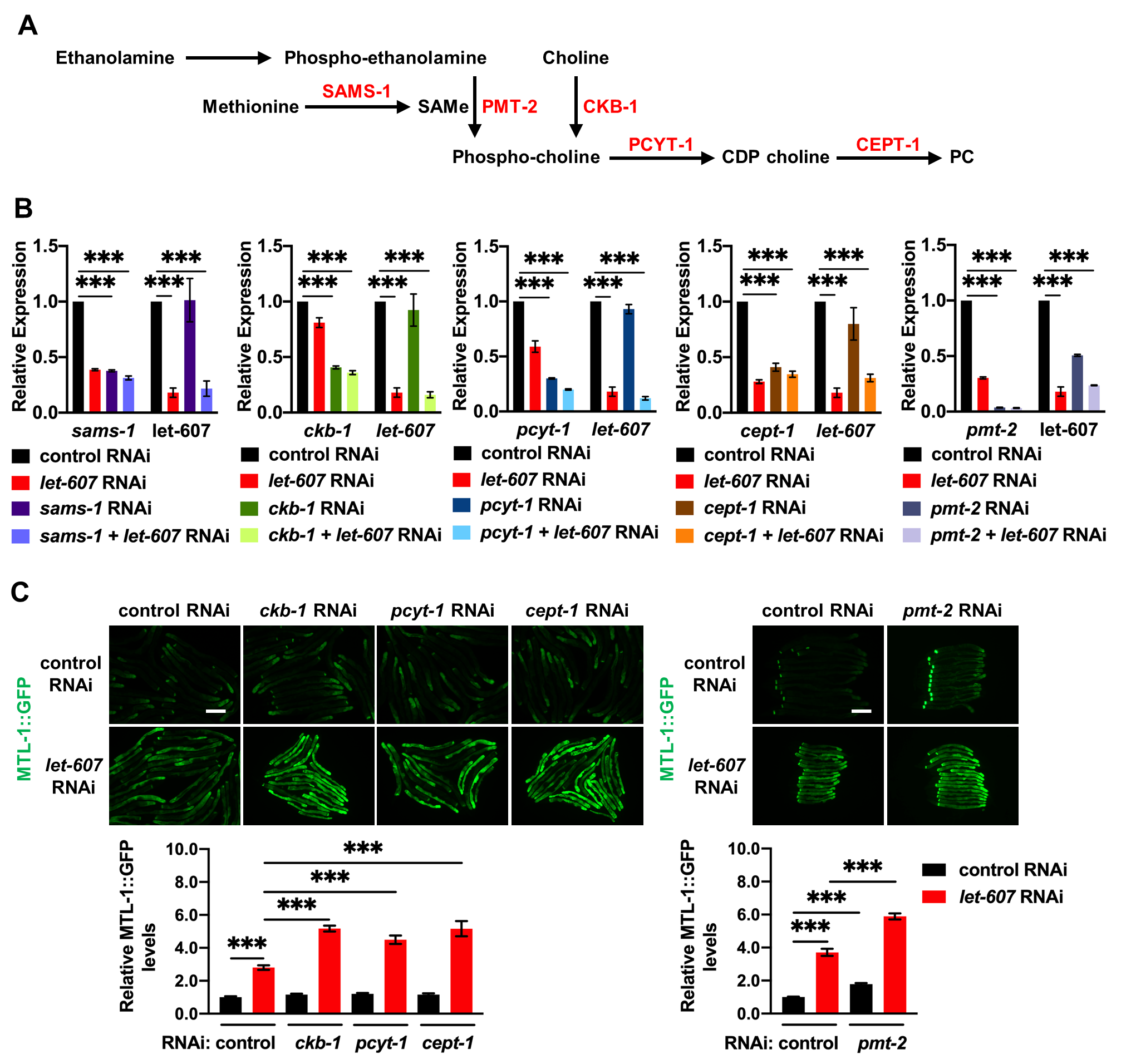

Supplement: S5 Fig — (A) The schematic of the PC biosynthetic pathway in C. elegans. (B) Knockdown efficiencies of PC biosynthetic genes RNAi. n = 3 per group. (C) Effects of PC biosynthetic genes RNAi on MTL-1::GFP expression in WT and let-607 knockdown animals. Upper panel, representative images. Lower panel: quantification data. Scale bar = 100 μm. Data were presented as mean ± SEM. *** p < 0.001. (TIF) [file pgen.1009573.s005.TIF]

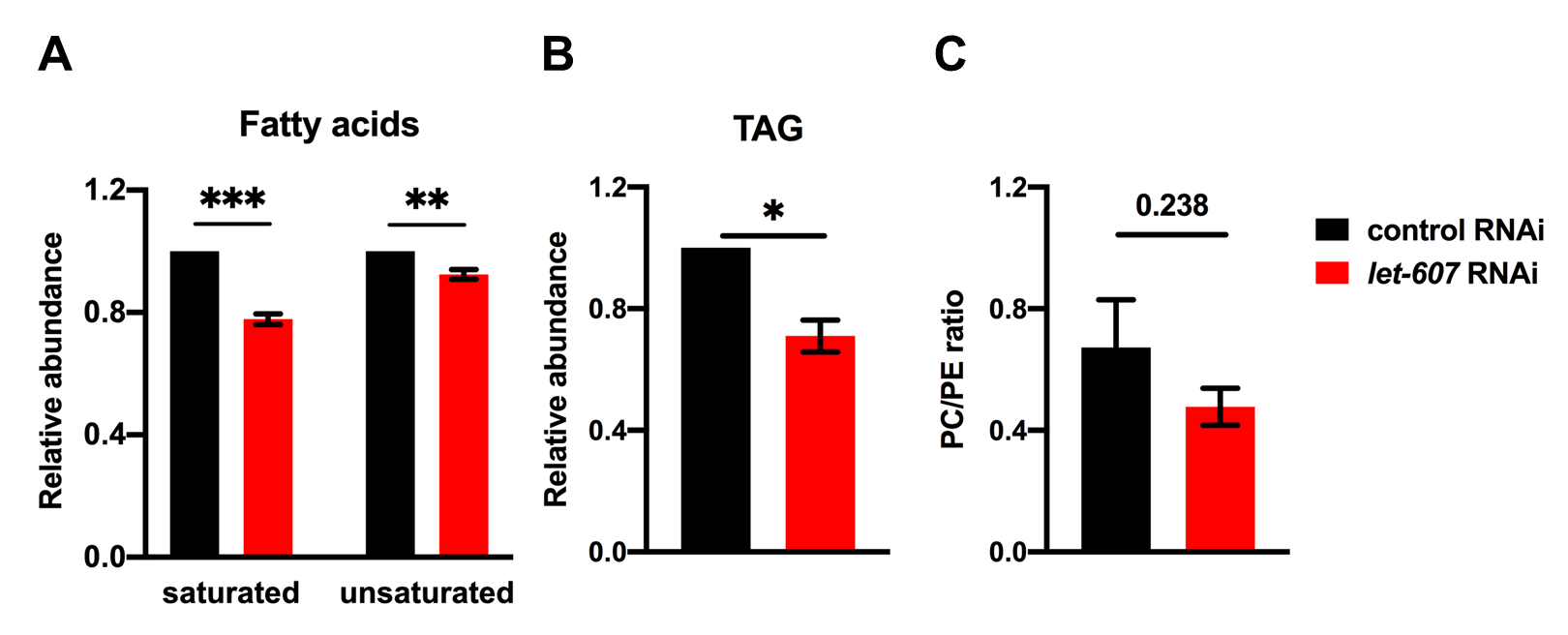

Supplement: S6 Fig — (A) Effects of let-607 RNAi on the abundance of total saturated and unsaturated fatty acids. (B) Total triglyceride levels after let-607 RNAi treatment. (C) PC/PE ratio after let-607 RNAi treatment. Data were presented as mean ± SEM. * p < 0.05, **p < 0.01, *** p < 0.001. (TIF) [file pgen.1009573.s006.TIF]

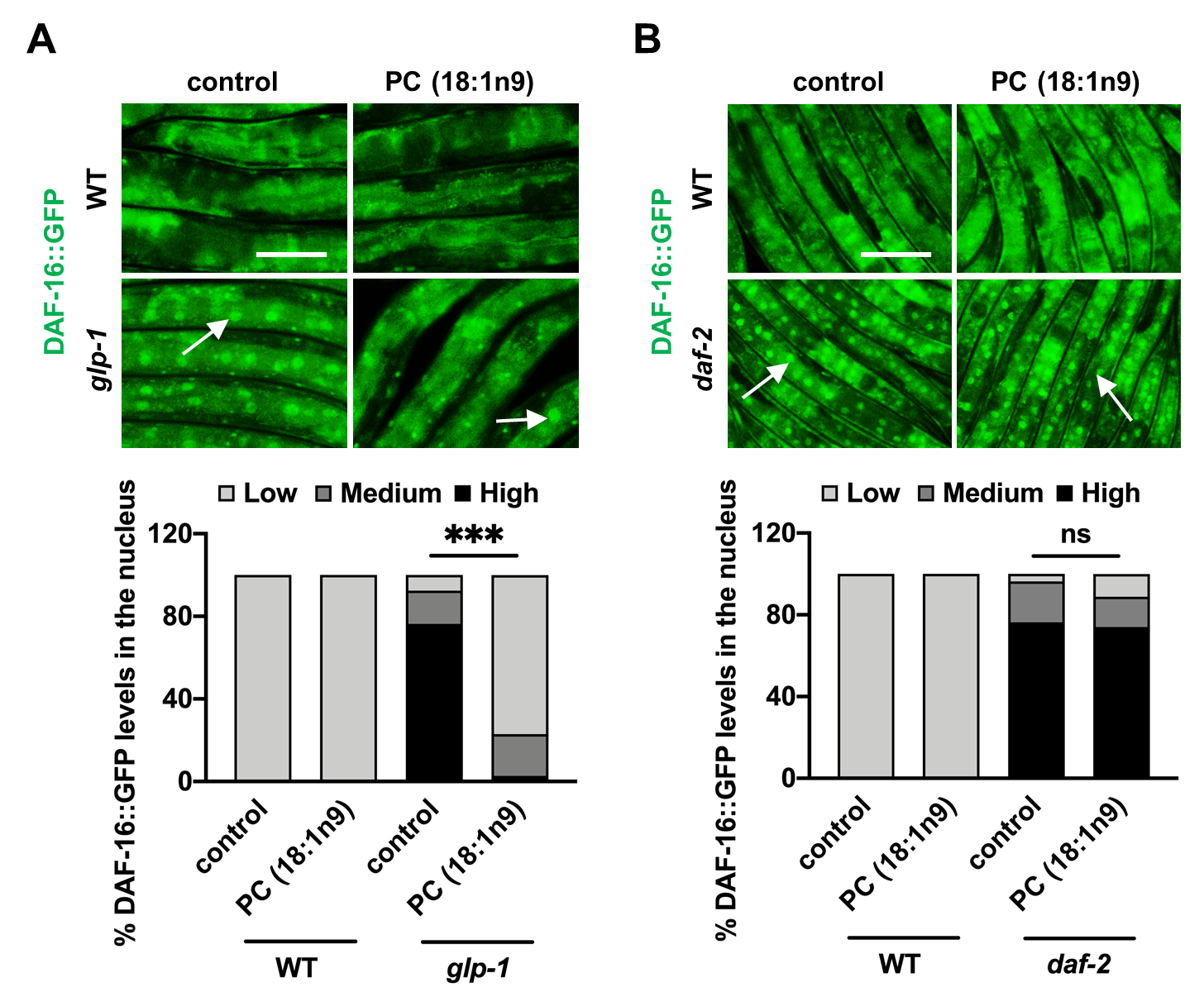

Supplement: S7 Fig — (A-B) Effects of PC (18:1n9) supplementation on DAF-16::GFP nuclear accumulation in glp-1 mutants (A) and daf-2 mutants (B). Scale bar = 100 μm. Upper panel shows representative images. White arrow indicates the nuclear GFP signal. Lower panel shows semi-quantification data. Number of animals (n) for (A): WT + control RNAi (85), WT+ let-607 RNAi (85), glp-1 + control RNAi (93), glp-1 + let-607 RNAi (109); number of animals (n) for (B): WT + control RNAi (65), WT+ let-607 RNAi (65), daf-2 + control RNAi (55) and daf-2 + let-607 RNAi (54). Scale bar = 50 μm. *** p < 0.001. (TIF) [file pgen.1009573.s007.TIF]

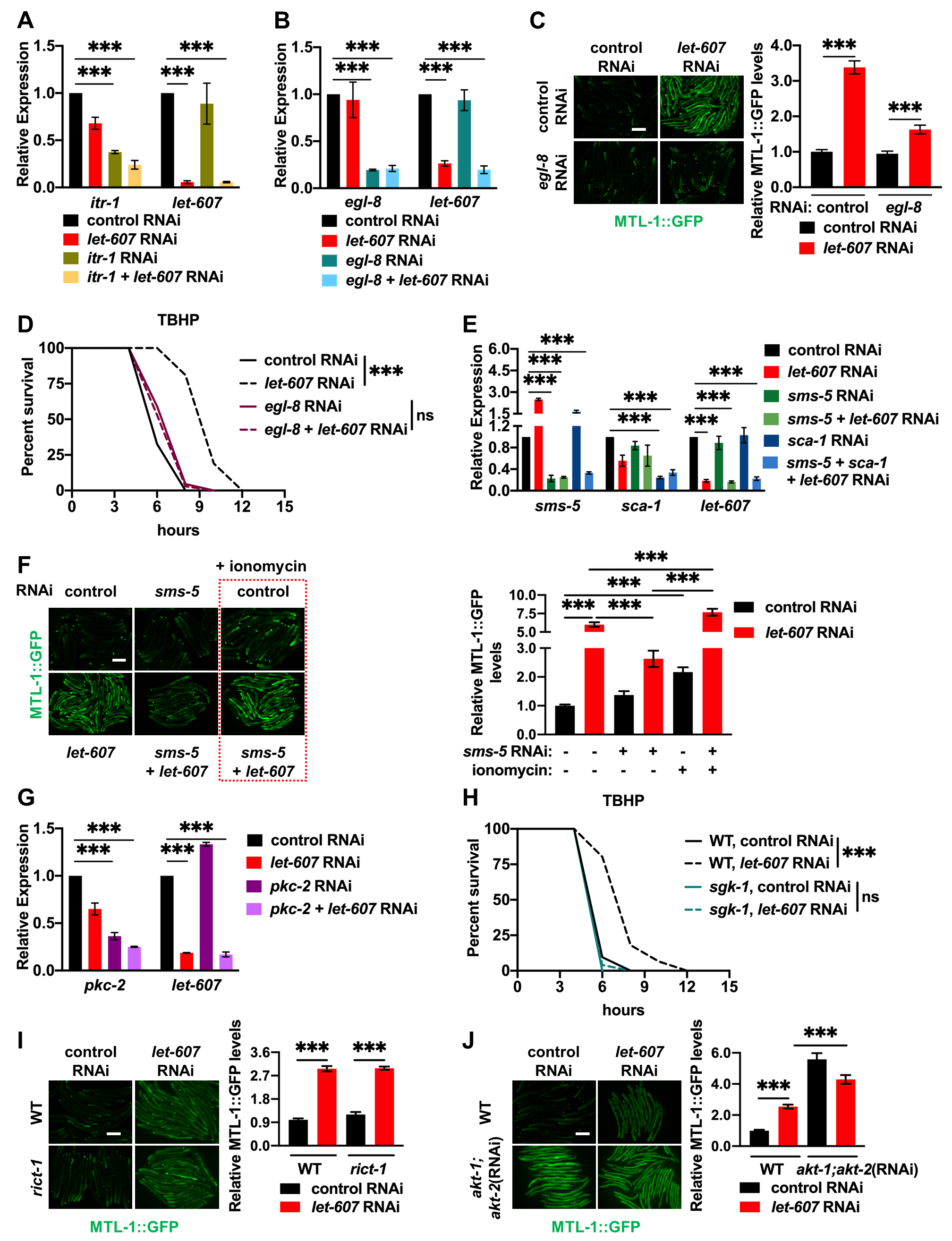

Supplement: S8 Fig — (A-B) Knockdown efficiencies of itr-1 RNAi (A) and egl-8 RNAi (B). (C-D) Effects of egl-8 RNAi on let-607 RNAi-induced MTL-1::GFP expression (C) and TBHP resistance (D). Left panel, representative images. Right panel: quantification data. Scale bar = 100 μm. (E) Knockdown efficiencies of sca -1 and sms-5 RNAi in Fig 7D. (F) Effects of ionomycin on MTL-1::GFP expression suppressed by sms-5 RNAi. Left panel, representative images. Right panel: quantification data. Scale bar = 100 μm. (G) Knockdown efficiencies of pkc-2 RNAi. (H) Effects of sgk-1 mutation on the TBHP resistance of WT and let-607 knockdown worms. (I-J) Effects of rict-1 (I) and akt-1;akt-2 (RNAi) (J) mutations on let-607 RNAi-induced MTL-1::GFP expression. Left panel, representative images. Right panel: quantification data. For (A), (B), (E) and (G), n = 3 per group. Data were presented as mean ± SEM. *** p < 0.001. (TIF) [file pgen.1009573.s008.TIF]
