# Supplementary material for: Phosphatidylcholine mediates the crosstalk between LET-607 and DAF-16 stress response pathways
Source: PLoS Genet. 2021 May 20;17(5):e1009573. doi: 10.1371/journal.pgen.1009573 (PMC8172019; doi:10.1371/journal.pgen.1009573)
Supplement: S2 Table — (DOCX) [file pgen.1009573.s010.docx]

Table S2. PA14 survival data. Repeats 1 are graphed in indicated Figures.

| Figures | Strain/Treatment | Mean Lifespan  ± SEM (days) | # Worms  Censored/Total | P value |
| --- | --- | --- | --- | --- |
| 1D  repeat 1 | control RNAi | 4.00 ± 0.18 | 0/35 |  |
|  | *let-607* RNAi | 4.97 ± 0.17 | 0/35 | <0.001 ^a^ |
| 1D  repeat 2 | control RNAi | 4.00 ± 0.18 | 0/35 |  |
|  | *let-607* RNAi | 4.97 ± 0.16 | 0/35 | <0.001 ^a^ |
| 1D  repeat 3 | control RNAi | 4.09 ± 0.15 | 0/35 |  |
|  | *let-607* RNAi | 4.94 ± 0.17 | 0/35 | <0.001 ^a^ |
| 1D  repeat 4 | control RNAi | 3.75 ± 0.13 | 0/36 |  |
|  | *let-607* RNAi | 4.46 ± 0.15 | 0/37 | <0.001 ^a^ |
| 1D  repeat 5 | control RNAi | 3.86 ± 0.11 | 0/36 |  |
|  | *let-607* RNAi | 4.49 ± 0.13 | 0/37 | <0.001 ^a^ |
| 3A repeat 1 | WT, control RNAi | 3.67 ± 0.12 | 0/36 |  |
|  | WT, *let-607* RNAi | 4.44 ± 0.13 | 0/36 | <0.001 ^a^ |
|  | *daf-16*, control RNAi | 2.89 ± 0.12 | 0/35 |  |
|  | *daf-16*, *let-607* RNAi | 3.11 ± 0.14 | 0/35 | 0.213 ^a^ |
| 3A repeat 2 | WT, control RNAi | 3.49 ± 0.12 | 0/35 |  |
|  | WT, *let-607* RNAi | 4.50 ± 0.13 | 0/36 | <0.001 ^a^ |
|  | *daf-16*, control RNAi | 3.00 ± 0.11 | 0/35 |  |
|  | *daf-16*, *let-607* RNAi | 3.06 ± 0.13 | 0/35 | 0.605 ^a^ |
| 3A repeat 3 | WT, control RNAi | 3.53 ± 0.11 | 0/36 |  |
|  | WT, *let-607* RNAi | 4.23 ± 0.16 | 0/35 | <0.001 ^a^ |
|  | *daf-16*, control RNAi | 3.00 ± 0.13 | 0/35 |  |
|  | *daf-16*, *let-607* RNAi | 3.06 ± 0.08 | 0/35 | 0.814 ^a^ |
| 3A repeat 4 | WT, control RNAi | 4.19 ± 0.16 | 0/36 |  |
|  | WT, *let-607* RNAi | 5.11 ± 0.19 | 0/35 | <0.001 ^a^ |
|  | *daf-16*, control RNAi | 3.51 ± 0.11 | 0/37 |  |
|  | *daf-16*, *let-607* RNAi | 3.63 ± 0.13 | 0/35 | 0.605 ^a^ |
| 3A repeat 5 | WT, control RNAi | 3.94 ± 0.17 | 0/35 |  |
|  | WT, *let-607* RNAi | 5.06 ± 0.18 | 0/36 | <0.001 ^a^ |
|  | *daf-16*, control RNAi | 3.44 ± 0.12 | 0/36 |  |
|  | *daf-16*, *let-607* RNAi | 3.57 ± 0.14 | 0/35 | 0.814 ^a^ |
| S3A repeat 1 | WT, control RNAi | 3.94 ± 0.17 | 0/35 |  |
|  | WT, *let-607* RNAi | 5.06 ± 0.18 | 0/36 | <0.001 ^a^ |
|  | *hsf-1*, control RNAi | 2.87 ± 0.12 | 0/39 |  |
|  | *hsf-1*, *let-607* RNAi | 3.57 ± 0.14 | 0/37 | <0.001 ^a^ |
| S3A repeat 2 | WT, control RNAi | 4.19 ± 0.16 | 0/36 |  |
|  | WT, *let-607* RNAi | 5.11 ± 0.19 | 0/35 | <0.001 ^a^ |
|  | *hsf-1*, control RNAi | 2.86 ± 0.12 | 0/37 |  |
|  | *hsf-1*, *let-607* RNAi | 3.51 ± 0.14 | 0/35 | <0.001 ^a^ |
| S3A repeat 3 | WT, control RNAi | 3.86 ± 0.15 | 0/36 |  |
|  | WT, *let-607* RNAi | 5.16 ± 0.17 | 0/37 | <0.001 ^a^ |
|  | *hsf-1*, control RNAi | 2.86 ± 0.11 | 0/36 |  |
|  | *hsf-1*, *let-607* RNAi | 3.49 ± 0.11 | 0/35 | <0.001 ^a^ |
| S3A repeat 4 | WT, control RNAi | 3.49 ± 0.12 | 0/35 |  |
|  | WT, *let-607* RNAi | 4.50 ± 0.13 | 0/36 | <0.001 ^a^ |
|  | *hsf-1*, control RNAi | 2.47 ± 0.10 | 0/36 |  |
|  | *hsf-1*, *let-607* RNAi | 3.03 ± 0.12 | 0/35 | <0.001 ^a^ |
| S3A repeat 5 | WT, control RNAi | 3.53 ± 0.11 | 0/36 |  |
|  | WT, *let-607* RNAi | 4.23 ± 0.16 | 0/35 | <0.001 ^a^ |
|  | *hsf-1*, control RNAi | 2.40 ± 0.10 | 0/35 |  |
|  | *hsf-1*, *let-607* RNAi | 3.11 ± 0.14 | 0/35 | <0.001 ^a^ |

^a^ vs same same strain + control RNAi
