# Supplementary material for: Phosphatidylcholine mediates the crosstalk between LET-607 and DAF-16 stress response pathways
Source: PLoS Genet. 2021 May 20;17(5):e1009573. doi: 10.1371/journal.pgen.1009573 (PMC8172019; doi:10.1371/journal.pgen.1009573)
Supplement: S3 Table — (DOCX) [file pgen.1009573.s011.docx]

Table S3. TBHP survival data. Repeats 1 are graphed in indicated Figures.

| Figures | | Strain/Treatment | Mean Lifespan  ± SEM (hours) | # Worms  Censored/Total | P value |
| --- | --- | --- | --- | --- | --- |
| 1E repeat 1 | | control RNAi | 10.00 ± 0.33 | 0/31 |  |
|  |  | *let-607* RNAi | 13.60 ± 0.19 | 0/35 | <0.001 ^a^ |
| 1E repeat 2 | | control RNAi | 8.93 ± 0.23 | 0/30 |  |
|  |  | *let-607* RNAi | 13.14 ± 0.25 | 0/35 | <0.001 ^a^ |
| 1E repeat 3 | | control RNAi | 9.14 ± 0.25 | 0/37 |  |
|  |  | *let-607* RNAi | 13.53 ± 0.41 | 0/34 | <0.001 ^a^ |
| S1E repeat 1 | | VP303, control RNAi | 6.14 ± 0.06 | 0/73 |  |
|  |  | VP303, *let-607* RNAi | 7.37 ± 0.15 | 0/63 | <0.001 ^a^ |
| S1E repeat 2 | | VP303, control RNAi | 6.03 ± 0.03 | 2/62 |  |
|  |  | VP303, *let-607* RNAi | 6.73 ± 0.16 | 0/49 | <0.001 ^a^ |
| S1E repeat 3 | | VP303, control RNAi | 6.27 ± 0.09 | 0/52 |  |
|  |  | VP303, *let-607* RNAi | 7.80 ± 0.16 | 0/59 | <0.001 ^a^ |
| S1G repeat 1 | | DCL569, control RNAi | 7.27 ± 0.22 | 0/33 |  |
|  |  | DCL569, *let-607* RNAi | 6.79 ± 0.17 | 0/33 | 0.088 ^a^ |
| S1G repeat 2 | | DCL569, control RNAi | 7.51 ± 0.14 | 0/37 |  |
|  |  | DCL569, *let-607* RNAi | 7.80 ± 0.09 | 0/49 | 0.081 ^a^ |
| S1G repeat 3 | | DCL569, control RNAi | 6.36 ± 0.13 | 0/33 |  |
|  |  | DCL569, *let-607* RNAi | 6.21 ± 0.10 | 0/38 | 0.358 ^a^ |
| S1I repeat 1 | | WM118, control RNAi | 7.41 ± 0.36 | 0/37 |  |
|  |  | WM118, *let-607* RNAi | 7.09 ± 0.31 | 0/35 | 0. 413 ^a^ |
| S1I repeat 2 | | WM118, control RNAi | 6.00 ± 0.00 | 0/43 |  |
|  |  | WM118, *let-607* RNAi | 6.05 ± 0.05 | 0/37 | 0.281 ^a^ |
| S1I repeat 3 | | WM118, control RNAi | 6.28 ± 0.12 | 0/36 |  |
|  |  | WM118, *let-607* RNAi | 6.11 ± 0.07 | 0/37 | 0.222 ^a^ |
| S1K repeat 1 | | NR222, control RNAi | 6.92 ± 0.27 | 0/37 |  |
|  |  | NR222, *let-607* RNAi | 7.08 ± 0.32 | 0/39 | 0.631 ^a^ |
| S1K repeat 2 | | NR222, control RNAi | 7.79 ± 0.31 | 0/38 |  |
|  |  | NR222, *let-607* RNAi | 7.79 ± 0.31 | 0/39 | 0.970 ^a^ |
| S1K repeat 3 | | NR222, control RNAi | 7.49 ± 0.20 | 0/35 |  |
|  |  | NR222, *let-607* RNAi | 7.31 ± 0.23 | 0/35 | 0.626 ^a^ |
| S1L repeat 1 | | control RNAi | 7.68 ± 0.20 | 5/49 |  |
|  |  | post-developmental  *let-607* RNAi | 10.32 ± 0.27 | 0/50 | <0.001 ^a^ |
| S1L repeat 2 | | control RNAi | 8.62 ± 0.18 | 0/39 |  |
|  |  | post-developmental  *let-607* RNAi | 10.50 ± 0.40 | 1/37 | <0.001 ^a^ |
| S1L repeat 3 | | control RNAi | 9.43 ± 0.18 | 0/42 |  |
|  |  | post-developmental  *let-607* RNAi | 11.84 ± 0.34 | 0/50 | <0.001 ^a^ |
| S1L repeat 4 | | control RNAi | 8.33 ± 0.21 | 0/54 |  |
|  |  | post-developmental  *let-607* RNAi | 10.83 ± 0.30 | 3/54 | <0.001 ^a^ |
| 3B repeat 1 | | WT, control RNAi | 8.93 ± 0.23 | 0/30 |  |
|  |  | WT, *let-607* RNAi | 13.14 ± 0.25 | 0/35 | <0.001 ^a^ |
|  |  | *daf-16*, control RNAi | 8.81 ± 0.21 | 0/32 |  |
|  |  | *daf-16*, *let-607* RNAi | 10.74 ± 0.26 | 0/35 | <0.001 ^a^, <0.001 ^b^ |
| 3B repeat 2 | | WT, control RNAi | 9.14 ± 0.25 | 0/37 |  |
|  |  | WT, *let-607* RNAi | 13.53 ± 0.41 | 0/34 | <0.001 ^a^ |
|  |  | *daf-16*, control RNAi | 8.68 ± 0.24 | 0/41 |  |
|  |  | *daf-16*, *let-607* RNAi | 10.34 ± 0.58 | 0/35 | <0.001 ^a^, <0.001 ^b^ |
| 3B repeat 3 | | WT, control RNAi | 10.00 ± 0.33 | 0/31 |  |
|  |  | WT, *let-607* RNAi | 13.60 ± 0.19 | 0/35 | <0.001 ^a^ |
|  |  | *daf-16*, control RNAi | 9.88 ± 0.26 | 0/33 |  |
|  |  | *daf-16*, *let-607* RNAi | 12.29 ± 0.35 | 0/35 | <0.001 ^a^, <0.001 ^b^ |
| S3B repeat 1 | | WT, control RNAi | 8.11 ± 0.25 | 0/35 |  |
|  |  | WT, *let-607* RNAi | 12.69 ± 0.40 | 2/35 | <0.001 ^a^ |
|  |  | *hsf-1*, control RNAi | 6.86 ± 0.17 | 0/35 |  |
|  |  | *hsf-1*, *let-607* RNAi | 8.86 ± 0.38 | 0/35 | <0.001 ^a^ |
| S3B repeat 2 | | WT, control RNAi | 8.65 ± 0.23 | 0/31 |  |
|  |  | WT, *let-607* RNAi | 14.51 ± 0.33 | 0/35 | <0.001 ^a^ |
|  |  | *hsf-1*, control RNAi | 6.91 ± 0.17 | 0/35 |  |
|  |  | *hsf-1*, *let-607* RNAi | 9.88 ± 0.44 | 0/34 | <0.001 ^a^ |
| S3B repeat 3 | | WT, control RNAi | 7.94 ± 0.26 | 0/35 |  |
|  |  | WT, *let-607* RNAi | 10.74 ± 0.35 | 0/35 | <0.001 ^a^ |
|  |  | *hsf-1*, control RNAi | 6.74 ± 0.16 | 0/35 |  |
|  |  | *hsf-1*, *let-607* RNAi | 8.06 ± 0.32 | 0/35 | <0.001 ^a^ |
| S4A repeat 1 | | control RNAi | 6.03 ± 0.03 | 0/58 |  |
|  |  | *let-607* RNAi | 11.24 ± 0.26 | 1/61 | <0.001 ^a^ |
|  |  | *sms-5* RNAi | 6.72 ± 0.15 | 0/47 |  |
|  |  | *sms-5* + *let-607* RNAi | 9.32 ± 0.19 | 0/47 | <0.001 ^a^, <0.001 ^b^ |
| S4A repeat 2 | control RNAi | 6.57 ± 0.14 | 0/56 |  | |
|  | *let-607* RNAi | 9.51 ± 0.32 | 0/41 | <0.001 ^a^ | |
|  | *sms-5* RNAi | 7.63 ± 0.20 | 6/43 |  | |
|  | *sms-5* + *let-607* RNAi | 9.62 ± 0.25 | 3/52 | <0.001 ^a^, <0.001 ^b^ | |
| S4A repeat 3 | | control RNAi | 7.24 ± 0.17 | 0/50 |  |
|  |  | *let-607* RNAi | 10.75 ± 0.32 | 0/59 | <0.001 ^a^ |
|  |  | *sms-5* RNAi | 7.52 ± 0.18 | 0/63 |  |
|  |  | *sms-5* + *let-607* RNAi | 8.82 ± 0.23 | 0/44 | <0.001 ^a^, <0.001 ^b^ |
| 7B repeat 1 | | WT, control RNAi | 6.69 ± 0.18 | 0/35 |  |
|  |  | WT, *let-607* RNAi | 10.34 ± 0.31 | 0/35 | <0.001 ^a^ |
|  |  | *itr-1*, control RNAi | 6.82 ± 0.24 | 0/34 |  |
|  |  | *itr-1*, *let-607* RNAi | 7.54 ± 0.27 | 0/35 | 0.069 ^a^, <0.001 ^b^ |
| 7B repeat 2 | | WT, control RNAi | 6.63 ± 0.16 | 6/35 |  |
|  |  | WT, *let-607* RNAi | 9.39 ± 0.32 | 5/35 | <0.001 ^a^ |
|  |  | *itr-1*, control RNAi | 6.69 ± 0.16 | 4/35 |  |
|  |  | *itr-1*, *let-607* RNAi | 6.97 ± 0.20 | 0/35 | 0.308 ^a^, <0.001 ^b^ |
| 7B repeat 3 | | WT, control RNAi | 7.28 ± 0.16 | 0/36 |  |
|  |  | WT, *let-607* RNAi | 10.65 ± 0.26 | 4/35 | <0.001 ^a^ |
|  |  | *itr-1*, control RNAi | 7.37 ± 0.16 | 0/35 |  |
|  |  | *itr-1*, *let-607* RNAi | 8.00 ± 0.18 | 0/35 | 0.012 ^a^, <0.001 ^b^ |
| S7D repeat 1 | | control RNAi | 6.65 ± 0.13 | 0/49 |  |
|  |  | *let-607* RNAi | 10.00 ± 0.20 | 0/37 | <0.001 ^a^ |
|  |  | *egl-8* RNAi | 7.27 ± 0.17 | 0/44 |  |
|  |  | *egl-8* + *let-607* RNAi | 7.12 ± 0.19 | 0/34 | 0.548 ^a^ |
| S7D repeat 2 | | control RNAi | 7.20 ± 0.17 | 0/40 |  |
|  |  | *let-607* RNAi | 9.00 ± 0.20 | 0/48 | <0.001 ^a^ |
|  |  | *egl-8* RNAi | 7.71 ± 0.16 | 6/42 |  |
|  |  | *egl-8* + *let-607* RNAi | 6.56 ± 0.13 | 3/61 | 0.342 ^a^ |
| S7D repeat 3 | | control RNAi | 6.14 ± 0.08 | 0/42 |  |
|  |  | *let-607* RNAi | 8.72 ± 0.26 | 0/50 | <0.001 ^a^ |
|  |  | *egl-8* RNAi | 6.57 ± 0.13 | 0/46 |  |
|  |  | *egl-8* + *let-607* RNAi | 6.09 ± 0.06 | 0/44 | 0.003 ^a^ |
| S7H repeat 1 | | WT, control RNAi | 6.19 ± 0.07 | 0/63 |  |
|  |  | WT, *let-607* RNAi | 8.10 ± 0.19 | 0/61 | <0.001 ^a^ |
|  |  | *sgk-1*, control RNAi | 6.00 ± 0.00 | 0/53 |  |
|  |  | *sgk-1*, *let-607* RNAi | 6.08 ± 0.06 | 0/49 | 0.1394 ^a^ |
| S7H repeat 2 | | WT, control RNAi | 6.43 ± 0.12 | 0/47 |  |
|  |  | WT, *let-607* RNAi | 7.80 ± 0.17 | 0/51 | <0.001 ^a^ |
|  |  | *sgk-1*, control RNAi | 6.20 ± 0.08 | 0/50 |  |
|  |  | *sgk-1*, *let-607* RNAi | 6.23 ± 0.08 | 0/62 | 0.8270 ^a^ |
| S7H repeat 3 | | WT, control RNAi | 6.17 ± 0.08 | 0/47 |  |
|  |  | WT, *let-607* RNAi | 7.89 ± 0.09 | 0/109 | <0.001 ^a^ |
|  |  | *sgk-1*, control RNAi | 6.00 ± 0.00 | 0/64 |  |
|  |  | *sgk-1*, *let-607* RNAi | 6.11 ± 0.06 | 0/55 | 0.0595 ^a^ |

^a^ vs same same strain + control RNAi

^b^ vs WT *let-607* RNAi
