# Supplementary material for: Phosphatidylcholine mediates the crosstalk between LET-607 and DAF-16 stress response pathways
Source: PLoS Genet. 2021 May 20;17(5):e1009573. doi: 10.1371/journal.pgen.1009573 (PMC8172019; doi:10.1371/journal.pgen.1009573)
Supplement: S4 Table — (DOCX) [file pgen.1009573.s012.docx]

Table S4. 35℃ heat shock data. Repeats 1 are graphed in indicated Figures.

| Figures | Strain/Treatment | Mean Lifespan  ± SEM (hours) | # Worms  Censored/Total | P value |
| --- | --- | --- | --- | --- |
| 1F repeat 1 | control RNAi | 7.37 ± 0.19 | 0/35 |  |
|  | *let-607* RNAi | 10.69 ± 0.28 | 0/35 | <0.001 ^a^ |
| 1F repeat 2 | control RNAi | 7.26 ± 0.16 | 0/35 |  |
|  | *let-607* RNAi | 9.60 ± 0.24 | 0/35 | <0.001 ^a^ |
| 1F repeat 3 | control RNAi | 7.00 ± 0.16 | 0/38 |  |
|  | *let-607* RNAi | 9.58 ± 0.16 | 0/43 | <0.001 ^a^ |
| S1D repeat 1 | VP303, control RNAi | 7.54 ± 0.13 | 0/39 |  |
|  | VP303, *let-607* RNAi | 9.71 ± 0.36 | 0/34 | <0.001 ^a^ |
| S1D repeat 2 | VP303, control RNAi | 7.58 ± 0.15 | 2/38 |  |
|  | VP303, *let-607* RNAi | 9.08 ± 0.20 | 0/52 | <0.001 ^a^ |
| S1D repeat 3 | VP303, control RNAi | 8.10 ± 0.15 | 0/41 |  |
|  | VP303, *let-607* RNAi | 10.43 ± 0.30 | 0/37 | <0.001 ^a^ |
| S1F repeat 1 | DCL569, control RNAi | 7.19 ± 0.17 | 0/32 |  |
|  | DCL569, *let-607* RNAi | 7.26 ± 0.16 | 0/38 | 0.748 ^a^ |
| S1F repeat 2 | DCL569, control RNAi | 9.00 ± 0.21 | 0/40 |  |
|  | DCL569, *let-607* RNAi | 9.38 ± 0.18 | 0/39 | 0.302 ^a^ |
| S1F repeat 3 | DCL569, control RNAi | 7.85 ± 0.08 | 0/41 |  |
|  | DCL569, *let-607* RNAi | 8.00 ± 0.19 | 0/37 | 0.393 ^a^ |
| S1H repeat 1 | WM118, control RNAi | 5.94 ± 0.06 | 0/34 |  |
|  | WM118, *let-607* RNAi | 5.95 ± 0.05 | 0/37 | 0.952 ^a^ |
| S1H repeat 2 | WM118, control RNAi | 6.97 ± 0.17 | 0/35 |  |
|  | WM118, *let-607* RNAi | 7.14 ± 0.17 | 0/35 | 0.476 ^a^ |
| S1H repeat 3 | WM118, control RNAi | 6.97 ± 0.16 | 0/37 |  |
|  | WM118, *let-607* RNAi | 6.86 ± 0.15 | 0/44 | 0.625 ^a^ |
| S1J repeat 1 | NR222, control RNAi | 8.32 ± 0.23 | 0/37 |  |
|  | NR222, *let-607* RNAi | 8.48 ± 0.27 | 0/33 | 0.652 ^a^ |
| S1J repeat 2 | NR222, control RNAi | 9.18 ± 0.29 | 0/39 |  |
|  | NR222, *let-607* RNAi | 9.88 ± 0.31 | 0/33 | 0.085 ^a^ |
| S1J repeat 3 | NR222, control RNAi | 8.67 ± 0.25 | 0/33 |  |
|  | NR222, *let-607* RNAi | 8.75 ± 0.21 | 0/32 | 0.906 ^a^ |
| S1M repeat 1 | control RNAi | 8.08 ± 0.20 | 3/59 |  |
|  | post-developmental  *let-607* RNAi | 10.64 ± 0.31 | 7/54 | <0.001 ^a^ |
| S1M repeat 2 | control RNAi | 7.61 ± 0.21 | 7/51 |  |
|  | post-developmental  *let-607* RNAi | 9.46 ± 0.27 | 8/56 | <0.001 ^a^ |
| S1M repeat 3 | control RNAi | 7.20 ± 0.18 | 13/45 |  |
|  | post-developmental  *let-607* RNAi | 8.77 ± 0.28 | 15/65 | <0.001 ^a^ |
| S1M repeat 4 | control RNAi | 8.49 ± 0.22 | 7/51 |  |
|  | post-developmental  *let-607* RNAi | 10.13 ± 0.33 | 1/52 | <0.001 ^a^ |
| S3C repeat 1 | WT, control RNAi | 8.60 ± 0.29 | 0/30 |  |
|  | WT, *let-607* RNAi | 10.71 ± 0.34 | 0/34 | <0.001 ^a^ |
|  | *daf-16*, control RNAi | 7.70 ± 0.17 | 0/33 |  |
|  | *daf-16*, *let-607* RNAi | 9.06 ± 0.25 | 0/32 | <0.001 ^a^, <0.001 ^b^ |
| S3C repeat 2 | WT, control RNAi | 9.35 ± 0.18 | 0/34 |  |
|  | WT, *let-607* RNAi | 13.94 ± 0.41 | 2/34 | <0.001 ^a^ |
|  | *daf-16*, control RNAi | 7.78 ± 0.10 | 0/36 |  |
|  | *daf-16*, *let-607* RNAi | 10.88 ± 0.49 | 0/32 | <0.001 ^a^, <0.001 ^b^ |
| S3C repeat 3 | WT, control RNAi | 8.76 ± 0.22 | 0/34 |  |
|  | WT, *let-607* RNAi | 11.12 ± 0.30 | 0/34 | <0.001 ^a^ |
|  | *daf-16*, control RNAi | 7.20 ± 0.18 | 0/35 |  |
|  | *daf-16*, *let-607* RNAi | 8.34 ± 0.29 | 0/35 | <0.001 ^a^, <0.001 ^b^ |
| S3D repeat 1 | WT, control RNAi | 8.76 ± 0.22 | 0/34 |  |
|  | WT, *let-607* RNAi | 11.12 ± 0.30 | 0/34 | <0.001 ^a^ |
|  | *hsf-1*, control RNAi | 8.29 ± 0.22 | 0/34 |  |
|  | *hsf-1*, *let-607* RNAi | 8.88 ± 0.28 | 0/34 | 0.060 ^a^, <0.001 ^b^ |
| S3D repeat 2 | WT, control RNAi | 9.60 ± 0.14 | 0/35 |  |
|  | WT, *let-607* RNAi | 13.16 ± 0.23 | 0/38 | <0.001 ^a^ |
|  | *hsf-1*, control RNAi | 9.20 ± 0.20 | 0/35 |  |
|  | *hsf-1*, *let-607* RNAi | 10.88 ± 0.32 | 0/43 | <0.001 ^a^, <0.001 ^b^ |
| S3D repeat 3 | WT, control RNAi | 9.53 ± 0.19 | 0/34 |  |
|  | WT, *let-607* RNAi | 14.97 ± 0.27 | 0/35 | <0.001 ^a^ |
|  | *hsf-1*, control RNAi | 9.37 ± 0.28 | 0/38 |  |
|  | *hsf-1*, *let-607* RNAi | 11.26 ± 0.50 | 0/38 | <0.001 ^a^, <0.001 ^b^ |

^a^ vs same same strain + control RNAi

^b^ vs WT *let-607* RNAi
