# Supplementary material for: Phosphatidylcholine mediates the crosstalk between LET-607 and DAF-16 stress response pathways
Source: PLoS Genet. 2021 May 20;17(5):e1009573. doi: 10.1371/journal.pgen.1009573 (PMC8172019; doi:10.1371/journal.pgen.1009573)
Supplement: S5 Table — (DOCX) [file pgen.1009573.s013.docx]

Table S5. DTT survival data. Repeats 1 are graphed in indicated Figures.

| Figures | Strain/Treatment | Mean Lifespan  ± SEM (hours) | # Worms  Censored/Total | P value |
| --- | --- | --- | --- | --- |
| 1G repeat 1 | control RNAi | 6.21 ± 0.20 | 3/56 |  |
|  | *let-607* RNAi | 8.89 ± 0.32 | 6/51 | <0.001 ^a^ |
| 1G repeat 2 | control RNAi | 6.62 ± 0.22 | 3/65 |  |
|  | *let-607* RNAi | 8.90 ± 0.29 | 5/56 | <0.001 ^a^ |
| 1G repeat 3 | control RNAi | 7.00 ± 0.18 | 6/59 |  |
|  | *let-607* RNAi | 9.43 ± 0.30 | 6/55 | <0.001 ^a^ |
| 1G repeat 4 | control RNAi | 6.07 ± 0.18 | 1/72 |  |
|  | *let-607* RNAi | 8.62 ± 0.26 | 11/70 | <0.001 ^a^ |

^a^ vs same same strain + control RNAi
