# Supplementary material for: Phosphatidylcholine mediates the crosstalk between LET-607 and DAF-16 stress response pathways
Source: PLoS Genet. 2021 May 20;17(5):e1009573. doi: 10.1371/journal.pgen.1009573 (PMC8172019; doi:10.1371/journal.pgen.1009573)
Supplement: S6 Table — (DOCX) [file pgen.1009573.s014.docx]

Table S6. Lifespan data. Repeats 1 are graphed in indicated Figures.

| Figures | Strain/Treatment | Mean Lifespan  ± SEM (days) | # Worms  Censored/Total | P value |
| --- | --- | --- | --- | --- |
| 1H repeat 1 | control RNAi | 16.39 ± 0.62 | 2/55 |  |
|  | *let-607* RNAi | 24.95 ± 0.97 | 0/55 | <0.001 ^a^ |
| 1H repeat 2 | control RNAi | 16.23 ± 0.62 | 1/55 |  |
|  | *let-607* RNAi | 25.09 ± 0.87 | 0/55 | <0.001 ^a^ |
| 1H repeat 3 | control RNAi | 15.89 ± 0.67 | 0/55 |  |
|  | *let-607* RNAi | 26.52 ± 0.87 | 3/55 | <0.001 ^a^ |
| 1H repeat 4 | control RNAi | 16.33 ± 0.65 | 0/55 |  |
|  | *let-607* RNAi | 25.38 ± 0.91 | 0/55 | <0.001 ^a^ |
| S1N repeat 1 | control RNAi | 16.63 ± 0.65 | 5/59 |  |
|  | post-developmental  *let-607* RNAi | 22.10 ± 0.78 | 6/64 | <0.001 ^a^ |
| S1N repeat 2 | control RNAi | 17.76 ± 0.59 | 5/63 |  |
|  | post-developmental  *let-607* RNAi | 20.66 ± 0.75 | 0/61 | <0.001 ^a^ |
| S1N repeat 3 | control RNAi | 18.42 ± 0.52 | 2/64 |  |
|  | post-developmental  *let-607* RNAi | 21.79 ± 0.74 | 3/60 | <0.001 ^a^ |
| S1N repeat 4 | control RNAi | 17.50 ± 0.58 | 3/63 |  |
|  | post-developmental  *let-607* RNAi | 21.75 ± 0.84 | 6/62 | <0.001 ^a^ |
| 3C repeat 1 | WT, control RNAi | 18.69 ± 0.99 | 11/58 |  |
|  | WT, *let-607* RNAi | 24.79 ± 1.08 | 2/55 | <0.001 ^a^ |
|  | *daf-16*, control RNAi | 13.85 ± 0.40 | 2/55 |  |
|  | *daf-16*, *let-607* RNAi | 15.05 ± 0.54 | 4/49 | 0.022 ^a^, <0.001 ^b^ |
| 3C repeat 2 | WT, control RNAi | 17.93 ± 0.85 | 10/60 |  |
|  | WT, *let-607* RNAi | 25.93 ± 1.09 | 1/53 | <0.001 ^a^ |
|  | *daf-16*, control RNAi | 13.29 ± 0.51 | 4/55 |  |
|  | *daf-16*, *let-607* RNAi | 14.80 ± 0.67 | 4/49 | 0.008 ^a^, <0.001 ^b^ |
| 3C repeat 3 | WT, control RNAi | 17.93 ± 0.60 | 6/61 |  |
|  | WT, *let-607* RNAi | 22.76 ± 0.96 | 4/60 | <0.001 ^a^ |
|  | *daf-16*, control RNAi | 13.59 ± 0.33 | 6/62 |  |
|  | *daf-16*, *let-607* RNAi | 15.41 ± 0.41 | 4/62 | <0.001 ^a^, <0.001 ^b^ |
| 3C repeat 4 | WT, control RNAi | 19.10 ± 0.62 | 8/57 |  |
|  | WT, *let-607* RNAi | 23.19 ± 0.86 | 5/59 | <0.001 ^a^ |
|  | *daf-16*, control RNAi | 13.86 ± 0.36 | 11/62 |  |
|  | *daf-16*, *let-607* RNAi | 15.17 ± 0.39 | 4/62 | 0.016 ^a^, <0.001 ^b^ |
| S2J repeat 1 | WT, control RNAi | 18.87 ± 0.57 | 0/60 |  |
|  | WT, *let-607* RNAi | 25.90 ± 0.97 | 0/60 | <0.001^a^ |
|  | *daf-2*, control RNAi | 33.63 ± 1.49 | 0/59 |  |
|  | *daf-2*, *let-607* RNAi | 39.10 ± 1.65 | 0/60 | 0.014 ^a^ |
| S2J repeat 2 | WT, control RNAi | 18.93 ± 0.58 | 0/60 |  |
|  | WT, *let-607* RNAi | 26.69 ± 0.77 | 0/61 | <0.001 ^a^ |
|  | *daf-2*, control RNAi | 33.00 ± 1.34 | 0/60 |  |
|  | *daf-2*, *let-607* RNAi | 40.27 ± 1.65 | 0/60 | <0.001 ^a^ |
| S2J repeat 3 | WT, control RNAi | 18.21 ± 0.70 | 0/57 |  |
|  | WT, *let-607* RNAi | 24.54 ± 1.00 | 0/59 | <0.001 ^a^ |
|  | *daf-2*, control RNAi | 31.70 ± 1.55 | 0/60 |  |
|  | *daf-2*, *let-607* RNAi | 36.98 ± 1.68 | 0/61 | 0.006 ^a^ |
| S2J repeat 4 | WT, control RNAi | 18.04 ± 0.61 | 0/56 |  |
|  | WT, *let-607* RNAi | 24.60 ± 1.02 | 0/57 | <0.001 ^a^ |
|  | *daf-2*, control RNAi | 31.56 ± 1.47 | 0/59 |  |
|  | *daf-2*, *let-607* RNAi | 37.69 ± 1.76 | 0/59 | 0.001 ^a^ |
| 3D repeat 1 | WT, control RNAi | 17.47 ± 0.63 | 3/50 |  |
|  | WT, *let-607* RNAi | 27.68 ± 0.92 | 4/54 | <0.001 ^a^ |
|  | *glp-1*, control RNAi | 34.67 ± 1.20 | 3/56 |  |
|  | *glp-1*, *let-607* RNAi | 34.27 ± 1.22 | 3/56 | 0.862 ^a^ |
| 3D repeat 2 | WT, control RNAi | 17.23 ± 0.67 | 3/54 |  |
|  | WT, *let-607* RNAi | 25.44 ± 0.99 | 3/55 | <0.001 ^a^ |
|  | *glp-1*, control RNAi | 33.01 ± 1.27 | 2/54 |  |
|  | *glp-1*, *let-607* RNAi | 34.60 ± 1.25 | 3/55 | 0.376 ^a^ |
| 3D repeat 3 | WT, control RNAi | 17.55 ± 0.51 | 2/51 |  |
|  | WT, *let-607* RNAi | 23.48 ± 0.98 | 3/52 | <0.001 ^a^ |
|  | *glp-1*, control RNAi | 30.20 ± 1.52 | 5/55 |  |
|  | *glp-1*, *let-607* RNAi | 34.01 ± 1.33 | 6/55 | 0.142 ^a^ |
| 3D repeat 4 | WT, control RNAi | 17.99 ± 0.65 | 8/55 |  |
|  | WT, *let-607* RNAi | 23.85 ± 1.02 | 6/57 | <0.001 ^a^ |
|  | *glp-1*, control RNAi | 30.62 ± 1.44 | 6/55 |  |
|  | *glp-1*, *let-607* RNAi | 31.59 ± 1.37 | 6/54 | 0.906 ^a^ |
| 3E repeat 1 | WT, control RNAi | 15.44 ± 0.54 | 2/66 |  |
|  | WT, *let-607* RNAi | 25.16 ± 1.01 | 5/60 | <0.001 ^a^ |
|  | *daf-9*, control RNAi | 13.30 ± 0.47 | 0/60 |  |
|  | *daf-9*, *let-607* RNAi | 19.67 ± 0.63 | 2/63 | <0.001 ^a^ |
| 3E repeat 2 | WT, control RNAi | 15.38 ± 0.51 | 0/68 |  |
|  | WT, *let-607* RNAi | 24.07 ± 0.87 | 6/62 | <0.001 ^a^ |
|  | *daf-9*, control RNAi | 12.50 ± 0.39 | 1/61 |  |
|  | *daf-9*, *let-607* RNAi | 18.78 ± 0.62 | 3/62 | <0.001 ^a^ |
| 3E repeat 3 | WT, control RNAi | 14.82 ± 0.50 | 1/69 |  |
|  | WT, *let-607* RNAi | 24.69 ± 0.92 | 7/62 | <0.001 ^a^ |
|  | *daf-9*, control RNAi | 12.27 ± 0.40 | 1/60 |  |
|  | *daf-9*, *let-607* RNAi | 18.07 ± 0.72 | 1/60 | <0.001 ^a^ |
| 3E repeat 4 | WT, control RNAi | 15.08 ± 0.53 | 0/63 |  |
|  | WT, *let-607* RNAi | 23.28 ± 1.09 | 2/60 | <0.001 ^a^ |
|  | *daf-9*, control RNAi | 13.05 ± 0.39 | 0/63 |  |
|  | *daf-9*, *let-607* RNAi | 19.07 ± 0.61 | 2/60 | <0.001 ^a^ |
| 3F repeat 1 | WT, control RNAi | 17.38 ± 0.49 | 1/62 |  |
|  | WT, *let-607* RNAi | 25.97 ± 0.95 | 5/65 | <0.001 ^a^ |
|  | *daf-12*, control RNAi | 13.81 ± 0.45 | 0/62 |  |
|  | *daf-12*, *let-607* RNAi | 19.82 ± 0.71 | 5/61 | <0.001 ^a^ |
| 3F repeat 2 | WT, control RNAi | 17.43 ± 0.49 | 0/63 |  |
|  | WT, *let-607* RNAi | 24.34 ± 0.87 | 3/63 | <0.001 ^a^ |
|  | *daf-12*, control RNAi | 13.82 ± 0.47 | 0/66 |  |
|  | *daf-12*, *let-607* RNAi | 20.00 ± 0.64 | 3/66 | <0.001 ^a^ |
| 3F repeat 3 | WT, control RNAi | 16.83 ± 0.48 | 0/63 |  |
|  | WT, *let-607* RNAi | 25.15 ± 0.84 | 4/65 | <0.001 ^a^ |
|  | *daf-12*, control RNAi | 14.44 ± 0.45 | 0/64 |  |
|  | *daf-12*, *let-607* RNAi | 20.76 ± 0.80 | 3/61 | <0.001 ^a^ |
| 3F repeat 4 | WT, control RNAi | 16.32 ± 0.52 | 2/65 |  |
|  | WT, *let-607* RNAi | 23.92 ± 0.94 | 3/63 | <0.001 ^a^ |
|  | *daf-12*, control RNAi | 14.10 ± 0.48 | 0/63 |  |
|  | *daf-12*, *let-607* RNAi | 18.54 ± 0.77 | 1/60 | <0.001 ^a^ |
| 3G repeat 1 | WT, control RNAi | 17.70 ± 0.46 | 1/60 |  |
|  | WT, *let-607* RNAi | 24.11 ± 0.87 | 7/62 | <0.001 ^a^ |
|  | *tcer-1*, control RNAi | 20.64 ± 0.72 | 1/60 |  |
|  | *tcer-1*, *let-607* RNAi | 27.36 ± 0.98 | 3/62 | <0.001 ^a^ |
| 3G repeat 2 | WT, control RNAi | 16.81 ± 0.51 | 1/63 |  |
|  | WT, *let-607* RNAi | 24.81 ± 0.92 | 4/61 | <0.001 ^a^ |
|  | *tcer-1*, control RNAi | 19.94 ± 0.75 | 0/62 |  |
|  | *tcer-1*, *let-607* RNAi | 26.67 ± 1.19 | 4/61 | <0.001 ^a^ |
| 3G repeat 3 | WT, control RNAi | 16.97 ± 0.49 | 2/66 |  |
|  | WT, *let-607* RNAi | 26.00 ± 0.92 | 3/62 | <0.001 ^a^ |
|  | *tcer-1*, control RNAi | 19.10 ± 0.70 | 0/60 |  |
|  | *tcer-1*, *let-607* RNAi | 29.84 ± 1.01 | 1/63 | <0.001 ^a^ |
| 3G repeat 4 | WT, control RNAi | 17.02 ± 0.51 | 0/63 |  |
|  | WT, *let-607* RNAi | 23.07 ± 0.88 | 3/65 | <0.001 ^a^ |
|  | *tcer-1*, control RNAi | 21.15 ± 0.70 | 0/66 |  |
|  | *tcer-1*, *let-607* RNAi | 27.21 ± 1.12 | 3/61 | <0.001 ^a^ |
| 3H repeat 1 | WT, control RNAi | 16.55 ± 0.44 | 5/60 |  |
|  | WT, *let-607* RNAi | 23.43 ± 0.85 | 4/60 | <0.001 ^a^ |
|  | *kri-1*, control RNAi | 15.37 ± 0.55 | 3/66 |  |
|  | *kri-1*, *let-607* RNAi | 22.77 ± 0.89 | 1/61 | <0.001 ^a^ |
| 3H repeat 2 | WT, control RNAi | 14.95 ± 0.49 | 2/61 |  |
|  | WT, *let-607* RNAi | 22.44 ± 0.90 | 2/61 | <0.001 ^a^ |
|  | *kri-1*, control RNAi | 13.27 ± 0.57 | 5/59 |  |
|  | *kri-1*, *let-607* RNAi | 21.76 ± 1.00 | 2/61 | <0.001 ^a^ |
| 3H repeat 3 | WT, control RNAi | 14.90 ± 0.48 | 3/65 |  |
|  | WT, *let-607* RNAi | 20.23 ± 1.00 | 3/64 | <0.001 ^a^ |
|  | *kri-1*, control RNAi | 13.37 ± 0.54 | 2/62 |  |
|  | *kri-1*, *let-607* RNAi | 21.23 ± 1.09 | 1/61 | <0.001 ^a^ |
| 3H repeat 4 | WT, control RNAi | 12.69 ± 0.51 | 0/61 |  |
|  | WT, *let-607* RNAi | 22.33 ± 1.00 | 2/62 | <0.001 ^a^ |
|  | *kri-1*, control RNAi | 14.20 ± 0.53 | 0/61 |  |
|  | *kri-1*, *let-607* RNAi | 21.23 ± 1.03 | 1/63 | <0.001 ^a^ |
| S4B repeat 1 | control RNAi | 15.89 ± 0.67 | 0/55 |  |
|  | *let-607* RNAi | 26.52 ± 0.87 | 3/55 | <0.001 ^a^ |
|  | *sms-5* RNAi | 17.38 ± 0.84 | 0/55 |  |
|  | *sms-5* + *let-607* RNAi | 23.26 ± 0.97 | 0/54 | <0.001 ^a^, 0.174 ^b^ |
| S4B repeat 2 | control RNAi | 16.23 ± 0.62 | 1/55 |  |
|  | *let-607* RNAi | 25.09 ± 0.87 | 0/55 | <0.001 ^a^ |
|  | *sms-5* RNAi | 18.69 ± 0.84 | 0/55 |  |
|  | *sms-5* + *let-607* RNAi | 23.28 ± 0.88 | 0/58 | <0.001 ^a^, 0.181 ^b^ |
| S4B repeat 3 | control RNAi | 16.39 ± 0.62 | 2/55 |  |
|  | *let-607* RNAi | 24.95 ± 0.97 | 0/55 | <0.001 ^a^ |
|  | *sms-5* RNAi | 18.37 ± 0.89 | 1/55 |  |
|  | *sms-5* + *let-607* RNAi | 23.12 ± 0.94 | 0/57 | <0.001 ^a^, 0.034 ^b^ |
| S4B repeat 4 | control RNAi | 16.33 ± 0.65 | 0/55 |  |
|  | *let-607* RNAi | 26.19 ± 0.83 | 3/55 | <0.001 ^a^ |
|  | *sms-5* RNAi | 17.27 ± 0.94 | 0/55 |  |
|  | *sms-5* + *let-607* RNAi | 23.60 ± 0.99 | 0/55 | <0.001 ^a^, 0.116 ^b^ |
| 7C repeat 1 | WT, control RNAi | 16.39 ± 0.62 | 2/55 |  |
|  | WT, *let-607* RNAi | 24.95 ± 0.97 | 0/55 | <0.001 ^a^ |
|  | *itr-1*, control RNAi | 13.79 ± 0.37 | 2/55 |  |
|  | *itr-1*, *let-607* RNAi | 17.16 ± 0.66 | 0/55 | <0.001 ^a^, <0.001 ^b^ |
| 7C repeat 2 | WT, control RNAi | 16.23 ± 0.62 | 1/55 |  |
|  | WT, *let-607* RNAi | 25.09 ± 0.87 | 0/55 | <0.001 ^a^ |
|  | *itr-1*, control RNAi | 13.71 ± 0.41 | 0/55 |  |
|  | *itr-1*, *let-607* RNAi | 17.38 ± 0.65 | 0/55 | <0.001 ^a^, <0.001 ^b^ |
| 7C repeat 3 | WT, control RNAi | 15.89 ± 0.67 | 0/55 |  |
|  | WT, *let-607* RNAi | 26.52 ± 0.87 | 3/55 | <0.001 ^a^ |
|  | *itr-1*, control RNAi | 13.85 ± 0.36 | 0/55 |  |
|  | *itr-1*, *let-607* RNAi | 17.93 ± 0.78 | 0/60 | <0.001 ^a^, <0.001 ^b^ |
| 7C repeat 4 | WT, control RNAi | 16.33 ± 0.65 | 0/55 |  |
|  | WT, *let-607* RNAi | 25.38 ± 0.91 | 0/55 | <0.001 ^a^ |
|  | *itr-1*, control RNAi | 14.15 ± 0.38 | 0/55 |  |
|  | *itr-1*, *let-607* RNAi | 17.60 ± 0.75 | 0/56 | <0.001 ^a^, <0.001 ^b^ |
| 7G repeat 1 | control RNAi | 16.39 ± 0.62 | 2/55 |  |
|  | *let-607* RNAi | 24.95 ± 0.97 | 0/55 | <0.001 ^a^ |
|  | *sgk-1* RNAi | 14.92 ± 0.44 | 2/55 |  |
|  | *sgk-1* + *let-607* RNAi | 15.16 ± 0.39 | 0/55 | 0.838 ^a^ |
| 7G repeat 2 | control RNAi | 16.23 ± 0.62 | 1/55 |  |
|  | *let-607* RNAi | 25.09 ± 0.87 | 0/55 | <0.001 ^a^ |
|  | *sgk-1* RNAi | 15.13 ± 0.38 | 0/55 |  |
|  | *sgk-1* + *let-607* RNAi | 14.91 ± 0.39 | 0/55 | 0.790 ^a^ |
| 7G repeat 3 | control RNAi | 15.89 ± 0.67 | 0/55 |  |
|  | *let-607* RNAi | 26.52 ± 0.87 | 3/55 | <0.001 ^a^ |
|  | *sgk-1* RNAi | 14.18 ± 0.47 | 0/55 |  |
|  | *sgk-1* + *let-607* RNAi | 14.62 ± 0.41 | 0/60 | 0.781 ^a^ |
| 7G repeat 4 | control RNAi | 16.33 ± 0.65 | 0/55 |  |
|  | *let-607* RNAi | 25.38 ± 0.91 | 0/55 | <0.001 ^a^ |
|  | *sgk-1* RNAi | 14.25 ± 0.49 | 0/55 |  |
|  | *sgk-1* + *let-607* RNAi | 13.96 ± 0.55 | 0/57 | 0.925 ^a^ |

^a^ vs same same strain + control RNAi

^b^ vs WT *let-607* RNAi
