# Supplementary material for: Phosphatidylcholine mediates the crosstalk between LET-607 and DAF-16 stress response pathways
Source: PLoS Genet. 2021 May 20;17(5):e1009573. doi: 10.1371/journal.pgen.1009573 (PMC8172019; doi:10.1371/journal.pgen.1009573)
Supplement: S7 Table — (DOCX) [file pgen.1009573.s015.docx]

Table S7. qPCR primer sequences

| Primers | Sequences |
| --- | --- |
| *snb-1* Forward | GCAAGTATTGGTGGAAGA |
| *snb-1* Reverse | ACGATGATGATAATAAGAATGAC |
| *let-607* Forward | CGGATCTGATTGGAATGGAC |
| *let-607* Reverse | GATGAGGGAGATGTGCTTG |
| *daf-16* Forward | AAGCCGATTAAGACGGAACC |
| *daf-16* Reverse | GTAGTGGCATTGGCTTGAAG |
| *sod-3* Forward | CCAACCAGCGCTGAAATTCAATGG |
| *sod-3* Reverse | GGAACCGAAGTCGCGCTTAATAGT |
| *sms-5* Forward | AGAACCTGCTTCACGCCT |
| *sms-5* Reverse | GACGCCCACCATAGCAGTAG |
| *mtl-1 Forward* | ATGGCTTGCAAGTGTGACTG |
| *mtl-1 Reverse* | CACATTTGTCTCCGCACTTG |
| *itr-1 Forward* | TTGGAACACTGGTGGCTA |
| *itr-1 Reverse* | GGAAGCTGCACAGATTTCAT |
| *sca-1 Forward* | GTCGGAGTCGTCGGAATG |
| *sca-1 Reverse* | GGCAGGCTTCAGATTGTTG |
| *cept-1 Forward* | GCACCGTCATGGGCTTAT |
| *cept-1 Reverse* | CTTGCGTCATGCTATCACAAC |
| *pkc-2 Forward* | GACCGTTTGTATTTCGTGAT |
| *pkc-2 Reverse* | TTTATATGCCCGTCTCGTT |
| *egl-8 Forward* | GAAAGTACGTGTCTGGAACT |
| *egl-8 Reverse* | GCCATGCGTGATCCATAT |
| *pek-1 Forward* | ATGGAGGATCTGACAGAACT |
| *pek-1 Reverse* | CTCAATTCCTCCTGATGAAGAG |
| *atf-4 Forward* | CCATTCCACCCCACAATAT |
| *atf-4 Reverse* | GGAAGTTGACATCGGAGTT |
| *eif-2α Forward* | CAACATTCAATCAGGAGAGTCT |
| *eif-2α Reverse* | CGTTTCGTGGACCTTCAAT |
| *atf-6 Forward* | GAATCACAAGAATCGACCTCT |
| *atf-6 Reverse* | CCCACATTTCCTGGTCAT |
| *pdi-1 Forward* | TGAAGATGGAGTCGCTCTTAT |
| *pdi-1 Reverse* | AGGTCTCCTCCGACGAT |
| *pdi-2 Forward* | GACACCACCTCCGATGAT |
| *pdi-2 Reverse* | TTGGGTGAGCTTCTCGT |
| *ire-1 Forward* | TGGAAACTCTATCATCAGCGT |
| *ire-1 Reverse* | CCACGTATTCACTTCAGGC |
| *hsp-4 Forward* | GAAACAGAATCACTCCATCAT |
| *hsp-4 Reverse* | CAGTGCTTGATGTCTTGTT |
| *xbp-1s Forward* | TGCCTTTGAATCAGCAGTGG |
| *xbp-1s Reverse* | ACCGTCTGCTCCTTCCTCAATG |
| *aha-1 Forward* | TTCCATGTACTGTGTCTGC |
| *aha-1 Reverse* | TTCACTGGCTTGAGGTTG |
| *gsk-3 Forward* | ACGCATTCTTTGATGAGCT |
| *gsk-3 Reverse* | GCTTGTCGTTGAAACTTCAC |
| *sams-1 Forward* | CGGATATGCAACCGACGA |
| *sams-1 Reverse* | GACCACAACAGTGTGAACG |
| *ckb-1 Forward* | TGCCGTGTACCCCAAAT |
| *ckb-1 Reverse* | TTCATAGTCGCCACCGC |
| *pmt-2 Forward* | ATAAGGTGACCGAGGGAC |
| *pmt-2 Reverse* | TCGGCGTTGCGAATAGT |
| *pcyt-1 Forward* | TCATGGGCATGCGAATCAAC |
| *pcyt-1 Reverse* | AGTGCCGAACACCATCGTAA |
