# Supplementary material for: Phosphatidylcholine mediates the crosstalk between LET-607 and DAF-16 stress response pathways
Source: PLoS Genet. 2021 May 20;17(5):e1009573. doi: 10.1371/journal.pgen.1009573 (PMC8172019; doi:10.1371/journal.pgen.1009573)
Supplement: Attachment — Submitted filename: point by point response.docx [file pgen.1009573.s016.docx]

**We thank the reviewers for their professional and insightful comments. According to their suggestions, we have performed additional experiments and edited the text to improve our manuscript.**

*Reviewer #1: In this study, He et al. identify that depletion of the let-607 transcription factor leads to activation of the stress-related transcription factor DAF-16, resulting in enhanced cytotoxic protection and lifespan extension. They term this induction of DAF-16 activity as the ER-to-cytosol stress pathways communication (ECSPC), based on the assumption that LET-607 is the C. elegans CREBH homolog, embedded in the ER membrane. They further show that let-607 depletion alters fatty acid metabolism, and specifically reduced the levels of unsaturated PC, which turned out to be important for DAF-16 activation under these conditions. The authors identified additional genes required for DAF-16 activation by let-607 depletion including the IP3 receptor itr-1, which releases Calcium stores from the ER to the cytoplasm and the calcium-dependent kinase PKC-2.

I find that the beneficial effects of let-607 inactivation on lifespan and stress resistance, the associated activation of DAF-16, and the alterations in lipid metabolism are all well established. Specifically, the regulation of DAF-16 by unsaturated PC is novel and of wide-interest. Based on these, I find this work interesting and novel.

That being said, the main take-home message conveyed by the authors is that this is a pathway that links between the cytosolic and the ER stress responses. Nevertheless, the connection to the ER is mainly based on the finding that its transcript levels rise upon ER stress and the annotation of let-607 as the C. elegans ortholog of CREBH (whose connection to the ER is well established). However, this statement is based on homology spanning only 19% of the protein, corresponding to the b-leucine zipper domain. I could not find any indication for the presence of a putative transmembrane domain in the LET-607 protein. Such a transmembrane domain would be essential for anchoring the protein to the ER. I understand that let-607 has been referred to as the C. elegans ortholog of CREBH in previous publications, but this still needs to be established, especially since this is the “major player” in this work. Thus, the authors should investigate the intercellular localization of LET-607. Is it indeed embedded in the ER or is it cytosolic/nuclear? If this is done by tagging the protein, be careful not to perturb any putative signal peptide. If this experiment does not demonstrate ER localization – the paper should be re-written accordingly. To clarify – this experiment is needed and should be included in the final manuscript whatever the result may be – to confirm the ER association of this protein or to prevent further confusion.*

**Response:** We thank the reviewer for this insightful comment. Via the construction of a GFP-fused translational reporter LET-607::GFP, we found that LET-607 was mainly localized in the nucleus, not on the ER membrane (Figure 1B). As the reviewer said, this may be caused by the difference between the transmembrane domains of LET-607 and CREBH (Figure S1B). Intriguingly, a previous study reported that the *Drosophila* ortholog of CREBH lacks a transmembrane domain (Traffic, 2013, 14:382), implying certain lineages may lost the transmembrane domain. Our findings suggest that the function of LET-607/CREBH appears to be conserved, with a key role being to control the immune-related gene expression. However, LET-607 may have additional function due to its nuclear localization. The manuscript was re-written to focus on the crosstalk between LET-607 and DAF-16 stress pathways, without emphasizing involvement of ER.

*Additional major comments:
1) Epistatic analysis demonstrated that let-607 and germline depletion might act in the same or similar pathway as the lifespan effects were not additive. Likewise, both treatments lead to nuclear localization of DAF-16 specifically in the intestine. The question is – are these similar pathways that converge downstream, or does let-607 activate the germline pathway? This should be addressed in 2 ways: 1) What is the state of the germline in let-607 RNAi treated animals? 2) The longevity of glp-1 animals depends on unique genes such as daf-9, daf-12, kri-1, tcer-1 etc. Are these genes required for lifespan extension by let-607? Please do these experiments using mutants and let-607 RNAi (not double RNAis).*
**Response:** We thank the reviewer this important suggestion. In the revised manuscript, we first examined *C. elegans* reproduction and found that *let-607* RNAi reduced the numbers of progeny (Figure S3F). Next, we examined the effect of *let-607* RNAi on longevity in *daf-9*, *daf-12*, *tcer-1* or *kri-1* mutants. Each of the mutant genes was critical for germline-deficient longevity. The results showed all the above genes were dispensable for lifespan extension in *let-607* RNAi animals (Figure 3E-3H), suggesting that *let-607* knockdown may not promote longevity via activation of the germline pathway.

*2) Does sms-5 RNAi/PC(18:1N9) supplementation interfere with other daf-16 activating pathways such as daf-2,glp-1, oxidative stress, heat shock?*
**Response:** We performed the experiments as the reviewer suggested. It was shown that PC addition specifically suppressed DAF-16 induction in *glp-1* mutants (Figure 6A-6D, S6A-S6B), which is consistent with the lifespan data. In addition, *sms-5* RNAi did not affect DAF-16 activation in any of the long-lived models (Figure 6E and 6F) or under stressed conditions (Figure S6E and S6F). These data suggest that PC regulates DAF-16 activity, but SMS-5 is not responsible for PC regulation in *glp-1* mutants.

*3) Some of the experiments are done with multiple RNAis. This is especially problematic for epistasis analysis. These should be repeated using mutants when possible, or to the very least with a comparison of RNAi efficiencies.*
**Response:** We thank the reviewer for this valuable suggestion. Mutants were used in the main figures when possible, including *sgk-1*, *daf-9*, *daf-12*, etc. Also, a comparison of RNAi efficiencies was examined for all double RNAi experiments. The RNAi efficiency data were included in the supplementary figures.

*Additional comments:
Page 8 – “LET-607, like its mammalian ortholog, could also be activated upon ER stress18” – activation has not been shown here, only increased transcript levels have been demonstrated.*
**Response:** We have edited the text to state it accurately. The new sentence reads: The mRNA expression of *let-607* is induced by ER stress (Line 90).

*Page 8 – “Gene functional classification analysis revealed that the LET-607-upregulated genes were enriched in stress responses” - I am not sure if stress responses are the best definition here. The explicit stress response GO categories did not show up in this analysis. Please word this more carefully.*
**Response:** We have edited the text to state it accurately. The new sentence reads: Functional analysis revealed these genes were enriched in immune and detoxification responses (Line 101-102).

*Page 10 – “as RNAis of none of the ER UPR genes (ire-1, pek-1 and atf-6) had any effects on MTL-1::GFP expression” – negative result. Better done with mutants rather than RNAi.*
**Response:** We thank the reviewer for this important concern. We verified RNAi effects by examination of knockdown efficiencies as well as suppression of canonical target genes. The results showed that RNAi targeting ER UPR genes could significantly suppress UPR targets (Figure S2D), but did not affect MTL-1::GFP expression (Figure 2D).

*Page 10 – “We compared the let-607 RNAi-induced genes with the DAF-16 class I targets, and found that 74 genes upregulated by let-607 RNAi (13.6%) belonged to the DAF-16 class I targets, which was significantly greater than that would be expected by chance7 (Figure S2E), further supporting that let-607 RNAi regulates the activity of DAF-16. ” Please comment whether these genes also contain LET-607 binding sites.*
**Response:** A previous study used ChIP-seq analysis to identify genes possessing LET-607 binding sites within promoter regions (*Development*, 2016, 143:3540). As the reviewer suggested, we compared the 74 overlapped genes with their findings and found only one gene contained LET-607 binding sites, suggesting that LET-607 does not regulate these genes directly and likely functions via DAF-16.

*Figure 2 – number of animals scored is not the number of biological repeats.*
**Response:** This mistake has been corrected in the revised manuscript.

*A Summary model would be helpful.*

**Response:** A Summary model has been graphed as Figure 8.

*Reviewer #2: This manuscript by He et al. describes heretofore unappreciated genetic pathway connecting suppression of the ER stress factor CREBH/LET-607 to activation of a FoxO-dependent stress resistance pathway mediated by reduction in phosphatidylcholine containing fatty acids. The authors term this pathway the ER-to-cytosol stress pathways communication (ECSPC). The survival data are generally robustly gathered and I appreciate the inclusion of ~4 biological replicates for each of the conditions tested. Some of the mechanistic work based upon simultaneous knockdown of multiple genes at once with RNAi require more rigorous proof. Additionally, stronger genetic evidence for the involvement of LET-607 and factors previously reported to act in a pathway with LET-607 should be provided. I am generally positive on the novelty of the findings, but the work requires more rigor to substantiate a solid enough mechanism to garner publication in PLoS Genetics.

Although intelligible, the manuscript is written in broken English and absolutely requires proofreading by a native English speaker. There are too many errors to enumerate here.*
**Response:** We apologize for language errors. We have carefully edited the text and also used a professional language-editing service to improve our manuscript.

*There is no mention of significance for the calculated enrichment score and what the comparator group is for the analysis conducted in Figure 1B. Also, I assume that what the authors are referring to in Fig 1B when they say “LET-607-upregulated genes” is the set of genes upregulated when LET-607 is knocked down by RNAi. The manuscript would be well served if this was made more clear.*
**Response:** We thank the reviewer for this suggestion. Gene functional classification analysis was used by Database for Annotation, Visualization, and Integrated Discovery (DAVID). First, genes with similar annotation terms were grouped together. Subsequently, the geometric mean (in -log scale) of EASE scores (p-value) of all enriched annotation terms in each group was calculated as enrichment score. We have included this information in the Materials and Methods section.

Worms treated with control RNAi (L4440) were used as a control group for RNA-seq analysis and this information has been added to the text (Line 98).

“LET-607-upregulated genes” are actually genes downregulated by *let-607* RNAi. We apologize for this misunderstanding and have added a sentence to make this clearer. The new sentence reads: Genes which were decreased by *let-607* RNAi were classified as upregulated by LET-607 (Line 100-101).

*Are there other genes that activate the ECSPC in concert with CREBH, including previously reported partners/activators in CREBH/LET-607 activity BMAL1/AHA-1, GSK3b/GSK-3, or PPARalpha/NHR-49? Does genetic inactivation of the S1P/S2P also activate the ECSPC?*
**Response:** As the reviewer suggested, we have tested the role of *aha-1*, *gsk-3* and *nhr-49* in DAF-16 induction upon *let-607* RNAi. However, none of these genes were found to be involved (Figure S2E-S2H). As new data suggest LET-607 is mainly localized in the nucleus, not on the ER membrane (Figure 1B), we did not test the S1P/S2P, which regulate CREBH cleavage on the Golgi.

*Does the nuclear form of CREBH/LET-607 act as a dominant suppressor of the ECSPC?*
**Response:** As new data suggest LET-607 is mainly localized in the nucleus, not on the ER membrane (Figure 1B), there is no nuclear specific form of LET-607.

*In addition to “n”, the tests used to calculate significance should be indicated in the main figure legends (for example but not limited to Figs. 2B, 2C), as well as significance for survival data (even if they are indicated with repeats in Table S2).*
**Response:** We thank the reviewer for this important suggestion. Statistic methods have been included in Figure legends and the significance for survival data has been added to the figures.

*It is possible that the doses of PC(16:0) and PC(18:0) were insufficient to raise C. elegans levels of these metabolites and that is the reason they “fail” to suppress daf-16 responses in figure 3E. The authors should measure abundances of these species in treated animals along with the successful supplementation of PC(18:1n9) to substantiate the claim that it is unsaturated PC that is mediating the signaling pathway. Certainly because more than just 18:1n9 fatty acids are changing in the PC fraction of let-607 RNAi treated animals in figure 4D, even though, as the authors point out, the majority of changes are seen in PC unsaturated fatty acid abundances.*
**Response:** We thank the reviewer for this professional concern. By examining lipid contents after PC supplementation, we found PC (18:1n9) was indeed absorbed and its contents were restored in *let-607* knockdown worms (Figure 5F). However, PC (16:0) and PC (18:0) supplementations did not change their contents significantly (See figures below). It is possible that worms can not uptake these saturated PC or these PC contents are strictly controlled by homeostatic mechanisms. As such, we have removed the data of saturated PC supplementation. PC (18:1n9) addition data was moved to new figure 5 to make it more logical. It was now shown that unsaturated PC contents (18:1n9) were reduced to activate DAF-16 upon *let-607* RNAi treatment (Figure 5E and 5F).

*While I believe that the authors are likely correct on the conclusion of the involvement of calcium in the ECSPC activation, more evidence is needed to solidify some of the lines of evidence. Specifically, for the double RNAi experiments, the authors are already diluting let-607 RNAi to prevent lethality, how do they know, for example in Figure 5A/D/E/F that this is because they are no longer effectively knocking down let-607? This is not proven by the nicely done qPCR in Figure S1A. Further, I don’t know what to think when three RNAi are combined—this is generally not accepted as standard practice in the field without proof of equally good knockdown (i.e.figure 5D).*
**Response:** We thank the reviewer for this concern. For original Figures 5A/D/E/F and other Figures involving double or triple RNAi, we have used qPCR to verify and compare the knockdown efficiencies. It was shown that RNAi efficiencies were comparable among groups. All these data have been included in the supplementary figures. For instance, in Figure 7D (original figure 5D), *let-607*, *sms-5* and *sca-1* were comparably knocked down in each group as measured by qPCR (Figure S7F).

*The demonstration of pkc-2 and sgk-1 dependency of the ECSPC are interesting and compelling, but the interpretation of these data are complicated. First, Sgk-1 mutants are viable, so consideration should be given to repeating the experiment in figure 5F with sgk-1 mutants, and not double RNAi. Second, while pkc-2 and sgk-1 are reported to be in a genetic pathway with each other with regard to cold survival in the worm, more canonical descending input into sgk-1 is through mTORC2. The authors should determine whether disruption of mTORC2 also recapitulates the sgk-1 result. Third, the best described input into daf-16 are the akt kinases. The authors should disrupt akt-1 and akt-2 by genetic mutation or RNAi and determine whether this mitigates activation of daf-16 in the ECSPC (e.g. in response to let-607 RNAi).*
**Response:** We have performed additional experiments as the reviewer suggested: First, *sgk-1* mutation has been used to verify the finding of *sgk-1* RNAi (Figure 7F and S7H). Second, a mutation of *rict-1*, a TORC2 component, was tested and found to not suppress MTL-1::GFP induction in *let-607* RNAi-treated worms (Figure S7I), suggesting TORC2 is not involved. Third, we suppressed AKT by combining *akt-1* mutation and *akt-2* RNAi [*akt-1;akt-2(RNAi)*] as previously reported. This was found to enhance MTL-1::GFP expression as expected. However, it had similar effects in both wild type and *let-607* RNAi animals (Figure S7J), suggesting that AKT is not relevant to LET-607-mediated DAF-16 activation. These data suggest PKC-2 and SGK-1 kinases are required for DAF-16 activation in response to *let-607* RNAi.

*Minor comments
Please describe the pathogen stress used in the figure legend (Fig. 1C) or the text, as well as more detail about the stresses (i.e. duration, dose) used in the legend for figures 1C-1G.*
**Response:** We have added the information of pathogen stress in the text (Line 114). The experimental detail about stress resistance assays have been included in the Materials and Methods section.

*Sphingomyelin on P.8 is misspelled.*

**Response:** This error has been corrected.
