# Supplementary material for: Phosphatidylcholine mediates the crosstalk between LET-607 and DAF-16 stress response pathways
Source: PLoS Genet. 2021 May 20;17(5):e1009573. doi: 10.1371/journal.pgen.1009573 (PMC8172019; doi:10.1371/journal.pgen.1009573)
Supplement: Attachment — Submitted filename: point by point response.docx [file pgen.1009573.s017.docx]

**We thank the reviewers for their comments and suggestions. We have modified our manuscript according to their suggestions.**

*Reviewer #1: I am overall pleased with the revised version of the manuscript, but still must insist on one follow-up experiment to clearly demonstrate the intra-cellular localization of LET-607.

I appreciate the attempts of the authors to determine let-607 localization by generating a translational fusion. However, the provided images in Fig 1B are of poor quality, of low resolution and lack markers. From the little I can see, I think the localization may be ER after all, rather than nuclear. Better imaging is required. Higher resolution, confocal imaging, contrast labelling of the nuclei and an ER marker to examine possible colocalization*
**Response:** We thank the reviewer for this important concern. We crossed the LET-607::GFP strain with DIS-3::mCHERRY worms. DIS-3 is a component of the nuclear RNA exosome complex that is enriched in the nucleoplasm but not in the nucleoli (***BioRxiv****,* *https://doi.org/10.1101/2020.11.23.395020*). It was identified that LET-607::GFP overlapped with DIS-3::mCHERRY in the nucleoplasm (new Fig 1B), suggesting the nuclear localization of LET-607. High resolution images were provided and text was edited accordingly (Page 5, Line 93-96). *On the same note, DAF-16::gfp images are also of poor quality (Fig 2C. Fig 4C). Please provide better images.*
**Response:** Better and enlarged images have been provided.

*Page 11, line 221 - since PC reduction alone did not activate DAF-16 - this sentence should be toned down.*

**Response:** The sentence has been edited and now read as “suggesting that PC reduction participates in the activation of DAF-16” (Page 12, Line 228-229).

*Page 12, line 250 - the suppression of mlt-1 expression in glp-1 mutants was only partial - tone down*

**Response:** The sentence has been edited and now read as “Supplementation of unsaturated PC (18:1n9) partially suppressed MTL-1::GFP induction in *glp-1* mutants” (Page 13, Line 258).

*Page 10, line 191 - Replace reproductivity with a better word*

**Response:** Reproduction was used to replace reproductivity.

*Page 14, line 271 - in defective in*

**Response:** The sentence has been edited and now read as “which results in defects in ER-derived calcium signaling” (Page 14, Line 279).

*Reviewer #2: This revised manuscript by He et al. for the most part addresses my concerns. I appreciate the addition of the data demonstrating interaction with the daf-2 and germlineless pathway, as well as the epistasis analyses with daf-9, daf-12, tcer-1 and kri-1. The PC-calcium pathway analyses are also compelling and well presented, though quantification of some of the reporter data would go a long way in strengthening the conclusions. It is an interesting story that is relevant to the broad readership of PLoS Genetics.

I have a few lingering concerns, mostly statistical with some minor points.

t-test is not appropriate for establishing significance when more than 1 test is being done, e.g. fig 2B where daf-16 and sod-3 expression are both tested—correction for multiple hypothesis testing is needed.

Paired student t-tests are not appropriate without multiple hypothesis testing for figures 5B, C, and F. B and C should be corrected as A and D for multiple hypothesis testing, and F should be compared by 2-way ANOVA with post-hoc tests for significance.*
**Response:** We thank the reviewer for these concerns and apologize for the mistakes on statistics. We have used the correct statistic methods as the review suggested and stated the information clearly in the methods section (Page 22-23).

*Minor points:
The authors use dilutional RNAi. Did they consider post-developmental RNAi to see if the lifespan extension and developmental lethality represent antagonistic pleiotropies?*
**Response:** We thank the reviewer for this suggestion and agree that the post-developmental data is important. We have done this experiment, but did not include it in the previous manuscript. The results showed that post-developmental treatment of *let-607* RNAi could still enhance stress resistance and extend lifespan (new Fig S1L-S1N), suggesting that the effects of LET-607 in development and longevity may represent antagonistic pleiotropy.

*Line 60: based upon their findings, the authors may want to say “In mammals, CREBH is an ER-bound transcription factor…”
Line 68 LET-607 “is located in the nucleus” or “localizes to the nucleus”
In figure S1B, membrane is spelled “membrance” in 2 places.*
**Response:** We apologize for the errors and have them all corrected in the revised manuscript.

*It may be useful, when making decisions about additivity of factors in stress pathway activation, to quantify data—e.g. fig S2f-H. Then again, is it really valuable to be making comments about additivity of hypomorphic effects by RNAi? A hypomorphic effect can be enhanced almost by definition. As such the analyses are not terribly informative as presented.*

*The authors may consider quantifying expression of mtl-1 to make conclusions about synthetic interactions in figs. 4 and S4.*

*The data in figure 6 are very interesting and while S6 is quantified, Fig 6 is not, especially in glp1 6A where a claim of dependence on PC is made. These data should be quantified.*

**Response:** We thank the reviewer for these valuable suggestions. We have quantified the MTL-1::GFP throughout the manuscript.

For double RNAi experiments, we ensured same dilutions for RNAi clones in each experiment set and verified comparable knockdowns for each gene. For major findings such as PC and calcium signaling involvement, additional assays like PC supplementation were used to strengthen the conclusion. We agree with the reviewer that for some negative data, e.g. Fig S2F and S2G, double RNAi data might not be conclusive, and the conclusion was toned down accordingly in the revised manuscript (Page 8, Line 156-157).

*Please state the statistical test used in Fig 2E.*

**Response:** The statistical test for the overlap between two groups of genes was performed by using software provided by Jim Lund, accessible at http://nemates.org/MA/progs/overlap_stats.html. This information was stated in the methods section in the revised manuscript (Page 23, Line 463-467).

*Sgk-1 RNAi has been reported to be long lived (Baumeister lab, 2004 Dev Cell). Can the authors explain why their RNAi is not long lived in fig. 7G?*

**Response:** The divergence between lifespan data may result from the different methods used. In the *Dev Cell* paper, they stated: *To determine mean adult life span at 25°C, animals were grown at 15°C until the L4 larval stage and then shifted to prevent dauer entry*. We performed the lifespan analysis at 20°C. Since the culture temperature during development has significant impact on *C. elegans* lifespan, such difference may explain the long-lived phenotype of *sgk-1* RNAi in their paper. Furthermore, *sgk-1* null mutants were reported to be short-lived by other labs (*Soukas* et al*.,* [*2009*](https://www.ncbi.nlm.nih.gov/pmc/articles/PMC3824081/#b39) *Genes Dev; Alam* et al*.,* [*2010*](https://www.ncbi.nlm.nih.gov/pmc/articles/PMC3824081/#b2) *Cell Metab; Chen* et al.*, 2013 Aging Cell; Xiao* et al.*, 2013 Cell*), which are consistent with our data that worms with *sgk-1* knockdown are slightly short-lived.
